# Untargeted metabolomics-based network pharmacology reveals fermented brown rice towards anti-obesity efficacy

**DOI:** 10.1038/s41538-024-00258-x

**Published:** 2024-03-30

**Authors:** Kaliyan Barathikannan, Ramachandran Chelliah, Annadurai Vinothkanna, Ragothaman Prathiviraj, Akanksha Tyagi, Selvakumar Vijayalakshmi, Min-Jin Lim, Ai-Qun Jia, Deog- Hwan Oh

**Affiliations:** 1 Agricultural and Life Science Research Institute, Kangwon National University, Chuncheon, 24341 Korea; 2 Saveetha School of Engineering, Saveetha (SIMATS) University, Tamil Nadu, 600124 India; 3 Department of Food Science and Biotechnology, College of Agriculture and Life Sciences, Kangwon National University, Chuncheon, 200-701 South Korea; 4 Kangwon Institute of Inclusive Technology (KIIT), Kangwon National University, Chuncheon, 24341 South Korea; 5 School of Life Sciences, Hainan University, 570228 Haikou, China; 6 Hainan General Hospital, Hainan Affiliated Hospital of Hainan Medical University, 570311 Haikou, China; 7 Department of Microbiology, Pondicherry University, Puducherry, 605014 India

**Keywords:** Agriculture, Applied microbiology

## Abstract

There is a substantial rise in the global incidence of obesity. Brown rice contains metabolic substances that can help minimize the prevalence of obesity. This study evaluated nine brown rice varieties using probiotic fermentation using *Pediococcus acidilacti* MNL5 to enhance bioactive metabolites and their efficacy. Among the nine varieties, FBR-1741 had the highest pancreatic lipase inhibitory efficacy (87.6 ± 1.51%), DPPH assay (358.5 ± 2.80 mg Trolox equiv./100 g, DW), and ABTS assay (362.5 ± 2.32 mg Trolox equiv./100 g, DW). Compared to other fermented brown rice and FBR-1741 varieties, UHPLC-Q-TOF-MS/MS demonstrated significant untargeted metabolite alterations. The 17 most abundant polyphenolic metabolites in the FBR-1741 variety and 132 putative targets were assessed for obesity-related target proteins, and protein interaction networks were constructed using the Cystoscope software. Network pharmacology analysis validated FBR-1741 with active metabolites in the *C. elegans* obesity-induced model. Administration of FBR-1741 with ferulic acid improved lifespan decreased triglycerides, and suppressed the expression of fat-related genes. The enhanced anti-obesity properties of FBR-1741 suggest its implementation in obesity-functional food.

## Introduction

Obesity is a chronic disease due to significant fat accumulation in the body. By 2025, 167 million people and children will be unhealthy due to obesity, according to the World Health Organization (WHO)^1^. International dietary guidelines encourage the consumption of whole grains, especially brown rice, to minimize the risk of obesity, cardiovascular diseases, and type 2 diabetes^2^. The development of bio-functional materials increases the concentrations of the following substances: bioactive substances, γ-aminobutyric acid (GABA)^3^, ferulic acid, tocotrienols, potassium, zinc, and amino acids. Brown rice (BR) is a nutrient-dense food abundant in antioxidants, minerals, and other beneficial compounds. Brown rice’s low glycemic index could be beneficial to overweight people. Brown rice decreases the risk of obesity, while eating the same white rice elevates blood sugar levels after meals.

Grains provide a good alternative to dairy because, in addition to other nutritional advantages, they benefit from probiotic organisms that survive and are impervious to bile. Numerous cutting-edge technologies, such as bioconversion via fermentation, have been implemented to enhance the qualities of BR. Probiotic fermentation is recognized for improving animal and plant products’ dietary, sensory, and functional qualities. Fermentation is the most common and cost-effective means of enhancing food bioactive compounds. Fermented rice products prevent harmful bacteria and enhance flavor, texture, and consistency. Bioconversion/biotransformation utilizing catalysts and whole microbial organisms have produced alcoholic beverages and food from earlier civilizations^4^. Cereal-based fermented foods are healthy because they include beneficial bacteria and vitamins. The fermented cereals make organic acids, bacteriocins, and volatile substances essential for food safety, shelf life, and flavor. This group of bacteria includes *Streptococcus* and *Corynebacterium* species, as well as *Limosilactobacillus fermentum*, *Lactiplantibacillus plantarum*, *Lactococcus lactis*, *Levilactobacillus brevis*, *Pediococcus* species, and *Leuconostoc* species^5^. Liquid fermentation accelerates the production of metabolites, which depend on microbe proliferation^6^. Compared to the enzymatic process, microbial fermentation is the preferred commercial methodology for synthesizing active molecules due to its low cost and minimal environmental impact^7^. Polyphenolic compounds found in fermented brown rice have several physiological effects, such as significantly reducing blood cholesterol levels^8^.

Metabolomics is an approach for analyzing and effectively detecting molecules in sophisticated biological or fermentation systems. In fermentation, secondary metabolites may alter bacterial growth and metabolism. The alterations in metabolites can be used to infer the function of metabolic networks. Recent research has used NMR and UHPLC-Triple-TOF-MS/MS to identify the metabolic profile and delve into the highly overlapped metabolites^9^. High-throughput metabolomics is a developing field of research that analyzes the spectrum of metabolites found in biological samples. The primary objective of the multiomics approach is to find biomarkers of disease and promising biological targets for highly efficient drug screenings. Multiomics analyses investigate the molecular linkages to disease using environmental factors like diets. Omics’ investigations in humans and animals provide information on multiple diseases. In recent research on metabolic disease, omics’ findings from human and animal models were compared to confirm their clinical significance^10^. Bioinformatics analysis has helped find genomic biomarkers accurately from a large number of candidates at a lower cost and in less time compared to wet-lab-based experimental approaches for disease evaluation, prediction, and interventions^11^. Network pharmacology is a novel and effective approach for investigating the pharmacological process and revealing the functions and behaviors of sophisticated biological systems. Differentially expressed genes (DEGs) and hub genes can be used to study disease-related signaling networks and processes to predict targeted drugs^12^. For almost three decades, molecular docking has been a significant method for drug discovery, leading to the identification and development of many new drugs. Docking studies investigating the atomic-level interactions between a small metabolite and a protein, characterize molecules at target protein-binding sites and support the understanding of human diseases at the cellular level^13,14^.

In biomedical and nutritional research, *Caenorhabditis elegans* is a very useful model organism because it has a short life cycle, a unique way of absorbing fatty acids, simple genetics that is similar to humans, and it is easy to make mutants with targeted deletions of long-chain fatty acid metabolizing genes. In clinical research, these are generally prohibited due to ethical considerations and are expensive and time-consuming^15^. The *C. elegans*, a multicellular model organism, is small, inexpensive, easy to cultivate, has short generation cycles, and is genetically customizable. In *C. elegans*, triggering 112 genes raises fat stores, while suppressing 305 genes diminishes fat^16^. The hypodermic and intestinal cells of *C. elegans* contain lipids, which are simple to stain. Lipid affinity dyes allow for the visualization of fat accumulation and the convenient identification of lipid droplets in *C. elegans*. Decreased fat levels are linked to silencing the genes *sbp-1*, *hosl-1*, *fat-4*, *fat-5*, *fat-6*, and *fat-7*, which code for delta-9 fatty acid desaturation enzymes^17^. Dietary restriction (DR) extends the lifespan of several animals. DAF-16 modulates the insulin/IGF-1 signaling pathway in *C. elegans*, promoting lifespan^18^. In *C. elegans*, several SFAs, MUFAs, and PUFAs, including ω-6 AA (20:4ω-6), ω-3 EPA (20:5ω-3), and monomethyl branched-chain fatty acids, are present, as in humans^19^.

In this work, we explore the possibility of using fermentation methods to enhance the bioactive components and antioxidants in brown rice in order to develop functional foods that prevent obesity. We investigated the in vitro lipase inhibition assay and antioxidant properties of FBR. In addition, a network pharmacology method was applied to the identification of obesity-related metabolites using the UHPLC Q-TOF MS/MS technique, which was used to discover FBR metabolites. Furthermore, a *C. elegans* obesity model could be employed to validate the effect of the metabolites and FBR-1741 components by observing changes in lipid metabolism and reducing an accumulation of fat.

## Results and discussion

### Effect of brown rice fermentation

The nine brown rice varieties were evaluated for *P. acetolactic* MNL5. FBR-1741 exhibited the highest pancreatic lipase inhibitory activity among the nine brown rice varieties at the 48 h optimized incubation time. Furthermore, the strongest TPC, TFC, and antioxidant activities were also observed. In earlier investigations, fermenting diverse substrates increased the bioactive chemicals and anti-obesity potential in the MNL5 strain. Fermentation stimulates the biochemical processes of bacteria, which break down the cell wall structurally, producing various bioactive metabolites^16^.

### Impact of FBR on lipase inhibition

**Fig. 1:**
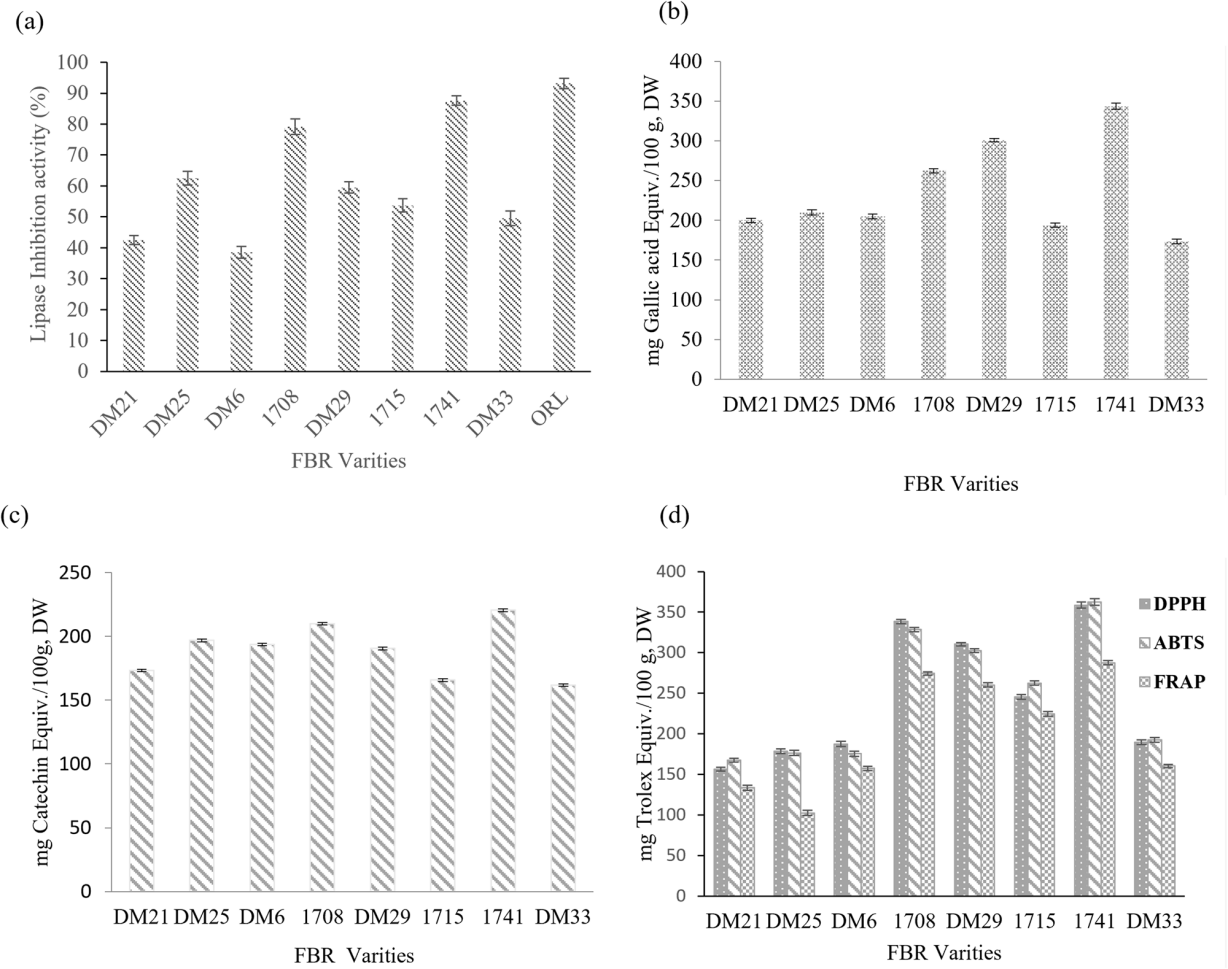
Anti-obesity in vitro pancreatic lipase inhibitory activity of fermented brown rice varieties. **a** Anti-obesity in vitro pancreatic lipase inhibitory activity of fermented Brown rice extracts. **b** Total phenolic content (TPC). **c** Total flavonoid content (TFC). **d** Antioxidant activity. Each bar represents the mean ± SD of triplicate values. 10.6084/m9.Figureshare.24781476.

The inhibitory activity of the pancreatic lipase enzyme and the associated compounds is shown in Fig. 1a, the fermentation process enhanced the lipase activity of brown rice compared to that of other rice varieties. Lipase inhibitory activity was highest in 1741 (87.6 ± 2.1%) > 1708 (79.2 ± 1.6%)>, while DM25 (62.5 ± 1.9%) and DM29 (59.5 ± 2.1%) showed enhancement. Lipase inhibition is an efficient technique for preventing the absorption of triacyl glycerides in people who suffer from hypercholesterolemia. Fermented materials suppress pancreatic lipase, although few reports have been published. Moreno et al.^20^ observed 80% lipase inhibition with 1 mg/mL grape seed extract. In addition, Wang et al.^21^ revealed that NTU 101-fermented green tea promotes lipase activity in adipocytes to limit lipid droplet formation and increase lipolysis.

### Total phenolic and flavonoid content of FBR

Figure 1b, c shows the TPC and TFC of nine varieties of fermented brown rice. TPC and TFC increased in FBR-1741 compared to the other FBR varieties. After comparison, FBR-1741 had the highest total phenolic and flavonoid content (TPC-343.5 ± 3.89 mg GAE equiv./100 g, DW; TFC-220.5 ± 3.81 mg GAE equiv./100 g). The second improved TPC and TFC content was FBR 1708 (Fig. 1b, c). The overall phenolic and flavonoid content is anticipated to benefit the antioxidant activity. Our findings suggest that more work is needed to boost the bioactivity of polyphenolics in FBRs and achieve antioxidant efficiency. Most cereals contain esterified connections to the matrix of the grain wall due to a lack of phenolic compounds^21^. Fermentation is a possible method for increasing the bioavailability of grain phenolics^22^. The above method may release insoluble or bonded phenolic substances. The current study compared nine varieties with fermentation using MNL5 and found that FBR-1741 enhanced polyphenols compared to other brown rice varieties from Korean resources.

### The effect of DPPH, ABTS, and FRAP scavenging activity

The enhanced antioxidant properties of brown rice extracts have been attributed to reducing capacity, metal ion chelation, and radical scavenging, among other mechanisms^23^. Absorption spectroscopy is a popular method for measuring the antioxidant activity of natural materials. FBR-1741 (358.5 ± 2.8 mg Trolox equiv./100 g, DW) and FBR-1708 (338.5 ± 3.1 mg Trolox equiv./100 g, DW) had the highest DPPH values. With the lowest results (156.5 ± 2.5 mg Trolox equiv./100 g, DW), FBR-DM21 was tested. Therefore, ABTS is necessary to assess the radical scavenging ability of grain. While the FRAP test was primarily designed to measure antioxidant capacity in plasma, it is now routinely used to analyze antioxidant capacity in isolated chemicals and biological materials^24^. It recognizes changes in absorbance brought on by blue iron (II) derived from iron oxide (III). The highest ABTS activity was observed in FBR-1741 (362.5 ± 3.3 mg Trolox equiv./100 g, DW). In addition, FBR-DM21 has the lowest ABTS activity. Similar results with DPPH were obtained in this study’s ABTS and FRAP tests. MNL5 FBR-1741 contained the maximum levels of FRAP (295.53.3 mg Trolox equiv./100 g, DW), followed by 1708 and DM21 (Fig. 1d). MNL5 FBR-1741 had the highest activity level out of the nine samples, according to the findings of the antioxidant assays. These results were greater than those reported previously by IIowefah et al.^25^ for fermented brown rice flour. The bioavailability of phenolics and flavonoids, as well as antioxidant activity, can be measured using this method, revealing variations in the extracts’ antioxidant activity. In addition, their chemical composition strongly influences the quenching power of the phenolic compounds. Antioxidants help prevent aging, obesity, and diabetes, which have been shown to be associated with the action of free radicals, as well as the degradation of essential fatty acids^26^. In recent years, fermentation has been regarded as an efficient method for enhancing the antioxidant activity of grains. Landete et al.^27^ reported the glycosidase activity of *L. plantarum* in relation to enhancing the bioaccessibility and bioavailability of dietary phenolic compounds and resulting in greater antioxidant activity. Our results show that FBR-1741, which was evaluated by DPPH, ABTS, and FRAP methods, has the strongest antioxidant activity.

### UHPLC-Q-TOF-MS/MS to identify the polyphenols in fermented brown rice

Our study used METLIN and the Metabolomics Workbench to assist us in determining the classification of polyphenolics. Table 1 shows that 17 phenolic substances were found in the ethanolic extracts of FBR-1741. This study obtained high amounts of phenolic substances formed in optimized conditions within 48 h. In addition, after 48 h of fermentation, ferulic acid, cinnamic acid, *p*-coumaric acid, protocatechuic acid, ethyl 4-hydroxybenzoate, caffeic acid, homovanillic acid, butylparaben, gallic acid, quercetin, isorhamnetin, sophoricoside, phenprobamate, daphnetin, cantharidin, genipin, and irisflorentin were identified. UHPLC-Q-TOF-MS/MS analysis identified 17 components in FBR, including 11 phonic compounds, four flavonoids, and two organic acids (Supplementary Fig. 1a–d). All of the top 17 active metabolites, especially ferulic acid, quercetin, isorhamnetin, irisflorentin, and protocatechuic acid, have a role in maintaining lipid metabolic stability. However, the FBR-1741 type exhibited the greatest level of phenolic compounds found. Based on our study, UHPLC-Q-TOF-MS/MS reveals the enhanced polyphenolic metabolites for FBR-1741 for anti-obesity functionality. In addition, 1708 shows 14 phenolic substances were found in the ethanolic extracts (Supplementary Table 1). Kuppusamy et al.^28^ observed that FA was imperative for antiadipogenic and lipogenic effects by regulating key adipocyte factors and enzymes and accelerating lipolysis via the HSL/perilipin mechanism. In addition, another investigation showed that ferulic acid reduced the risk of NAFLD and was developed as a functional food or beneficial substance^28^. In our prior study, fermented onion lowered cholesterol and enhanced quercetin to improve pancreatic lipase activity for synergistic anti-obesity benefits^16^. Isorhamnetin suppressed body fat and enhanced fat oxidation in the NHR-49-dependent pathway by lowering fat accumulation in a *C. elegans* study^29^. Jung et al.^30^ found that caffeic acid may reduce hyperglycemia by stimulating insulin secretion and reducing insulin resistance in db/db mice. Quercetin also improved the insulin resistance index, antioxidant enzyme activity, and insulin signaling in high-fat-diet-fed animals, showing its free-radical scavenging properties^31^.

**Table. 1:** Metabolites identified in FBR 1741 by UHPLC-Q-TOF-MS2

Sl. no.	RT (min)	Tentative compounds	Molecular formula	Precursor mass	Found at mass	Area	Adduct/charge
1.	16.12	Ferulic acid	C_10_H_10_O_4_	194.1800	194.0510	6.50E + 06	[M-H]–
2.	14.16	Cinnamic acid	C_9_H_8_O_2_	148.05308	147.0452	3.80E + 06	[M-H]–
3.	11.7	Protocatechuic acid	C_7_H_6_O_4_	154.02694	153.0198	2.10E + 06	[M-H]–
4.	6.17	*p*-Coumaric acid	C_9_H_8_O_3_	164.04775	163.0404	8.40E + 05	[M-H]–
5.	14.16	Ethyl 4-hydroxybenzoate	C_9_H_10_O_3_	166.06345	165.0557	8.90E + 06	[M-H]–
6.	10.59	Caffeic acid	C_9_H_8_O_4_	180.04288	179.0352	7.70E + 04	[M-H]–
7.	6.18	Homovanillic acid	C_9_H_10_O_4_	182.05830	181.0509	3.40E + 06	[M-H]–
8.	20.01	Butylparaben	C_11_H_14_O_3_	194.09510	193.0872	3.80E + 05	[M-H]–
9.	4.98	Gallic acid	C_11_H_12_N_2_O_2_	170.1256	169.0820	7.80E + 05	[M-H]–
10.	18.84	Quercetin	C_15_H_10_O_7_	302.04249	301.0346	2.90E + 06	[M-H]–
11.	19.65	Isorhamnetin	C_16_H_12_O_7_	316.05840	315.0508	1.10E + 06	[M-H]–
12.	17.19	Sophoricoside	C_21_H_20_O_10_	432.10629	431.0981	7.40E + 03	[M-H]–
13.	3.44	Phenprobamate	C_9_H_11_NO_2_	165.07949	164.0719	1.70E + 03	[M-H]–
14.	16.43	Daphnetin	C_9_H_6_O_4_	178.02734	177.0194	4.40E + 05	[M-H]–
15.	15.00	Cantharidin	C_10_H_12_O_4_	196.07429	195.0664	3.30E + 05	[M-H]–
16.	15.77	Genipin	C_11_H_14_O_5_	226.08458	225.0768	4.80E + 05	[M-H]–
17.	6.19	Irisflorentin	C_20_H_18_O_8_	386.09846	385.091	2.80E + 05	[M-H]–

*RT* retention time. 10.6084/m9.Figureshare.24781512

### Network pharmacology relations for FBR-1741 metabolites

**Fig. 2:**
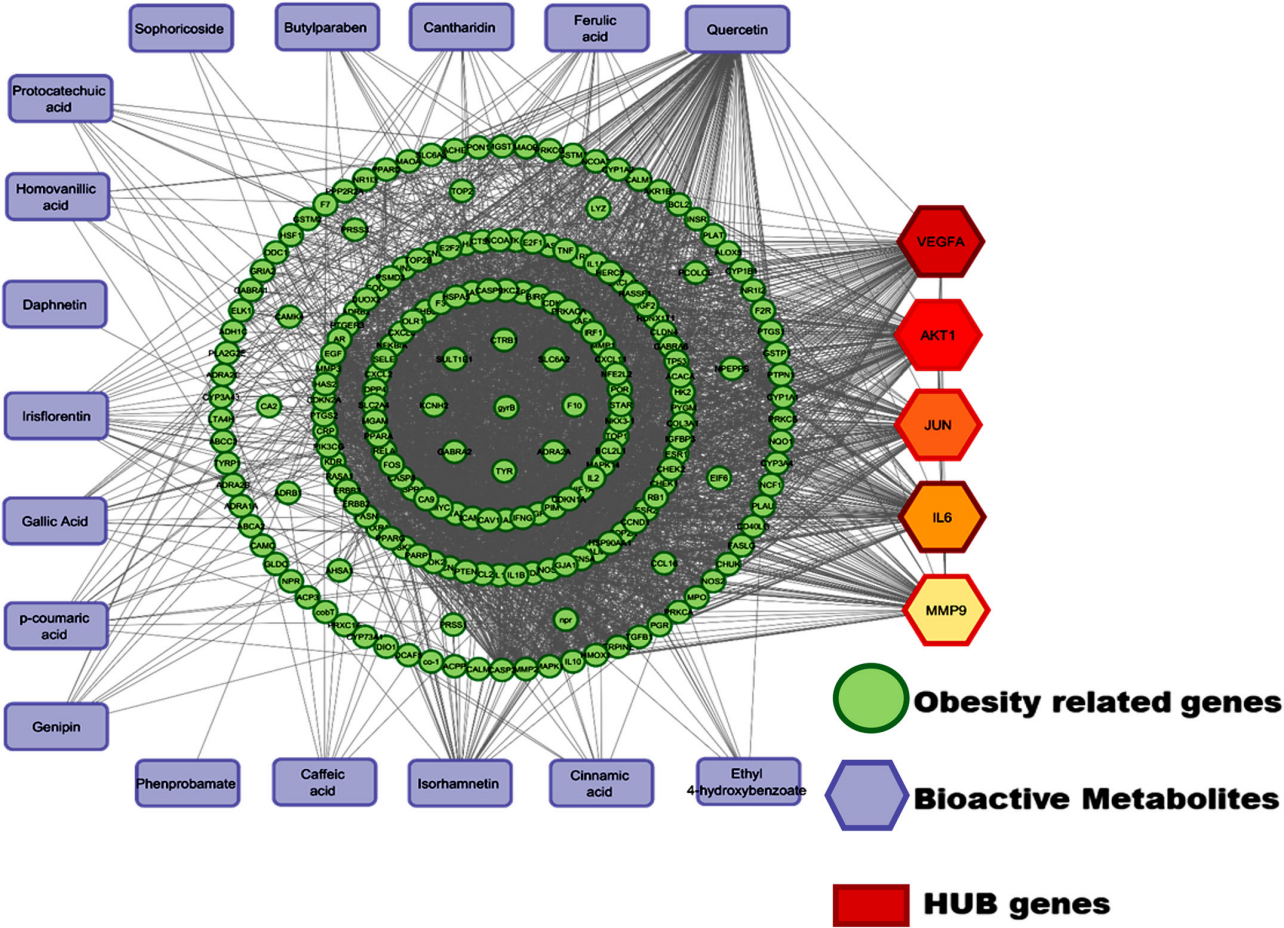
Target protein interaction network of 17 target metabolites in fermented brown rice for treating obesity-related diseases. The blue table nodes represent the 17 components, the red diamond represents the hub-gene, and the green circle nodes represent the corresponding 132 target genes of the ingredients. 10.6084/m9.Figureshare.24781479.

In the present study, 132 common target genes for obesity were acquired from available databases such as TCMSP, DisGeNET, and GeneCards (Supplementary Fig. 1a). The obesity-associated target genes interaction with 17 active metabolites chosen from FBR is provided in Supplementary Table 2. Drug screening criteria parameters, including OB, DL, and BBB, were selected for these 17 active compounds. The obesity-related target gene (132) interaction network was constructed using the STRING database with a high confidence score (0.700) (Supplementary Fig. 1b). The major hub proteins are vital in controlling and expressing all interacting sub-proteins to carry out their biological and molecular functions at any time. Five top-ranked hub proteins (VEGFA, AKT1, JUN, IL-6, and MMP9) were associated with the target disease–gene interaction network (Supplementary Fig. 1c). These selected hub proteins may be controlled/regulated by the biological and molecular functions of the remaining disease-associated target genes. According to the DisGeNET and GeneCards database analyses, five compounds showed the best drug-likeness networking properties against the five hub proteins. The bioactive FBR compounds (17) interacted with five hub proteins and the remaining disease-associated target genes. Among the 17 FBR compounds, ferulic acid, quercetin, isorhamnetin, protocatechuic acid, and irisflorentin had significant interactions with the top five hub proteins (Fig. 2). These results indicated that these FBR compounds might regulate or influence the genes related to obesity. Authenticating the hypothesis that VEGF-A concentration in serum is strongly associated with body mass index, elevated levels of VEGF-A were detected in humans as well as animal models that have become overweight or obese^32^. Adipose-VEGF stimulation reverses a cascade of events—adipocyte death, hypoxia, inflammatory processes, abnormal fat accumulation, and lipotoxicity—in chronically confronted animals and maintains glucose balance. According to Robciuc et al.^33^, inhibiting VEGFR-2 (KDR gene-encoded protein) activation might lower obesity by inhibiting angiogenesis and reducing fat mass. Shearin et al.^34^ showed that AKT1 signaling downstream of insulin and IGF-1 receptors regulates fat formation and regulation. IL-6 is an inflammatory factor that may predict insulin resistance and cardiovascular disease and contribute to their development. It is connected to abdominal adipose tissue and may influence TNF-α and other inflammatory factors. IL-6 and TNF-α are known to be produced by adipose tissue and may contribute to body mass augmentation^35^. According to Wang et al.^36^, network pharmacology-guided baicalin may reduce obesity by upregulating SLC2A1 and downregulating TNF, NFKB1, SREBF1, PPRGA, and CASP3. The current work focuses on the strong connections that VEGFA, AKT1, JUN, IL-6, and MMP9 hub genes for obesity-related functions have with the FBR-1741 metabolites.

### Performance of molecular docking

The five active FBR compounds were subjected to molecular docking with the top hub proteins. These compounds demonstrate the highest interaction with hub proteins. The binding scores, amino acid residues, and hydrogen bond distances are represented in Supplementary Table 3. Quercetin demonstrated the highest binding scores against the AKT1 (−6.8), IL6 (−7.2), and MMP9 (−8.0) hub proteins. Three amino acid residues, A: ARG169, A: MET68, and A: GLU173, with hydrogen bond distances of 2.544, 2.304, and 2.758 Å, respectively, were found against IL6. Isorhamnetin shows better interaction scores against the VEGFA (−8.5) and JUN (−5.3) hub genes than the other FBR compounds. Four hydrogen bond interactions with a strong h-bond distance were found against VEGFA (C: TYR357–2.243 Å; C: HIS415–2.059 Å; B: TYR299–2.918 Å and B: ILE418–2.406 Å). Irisflorentin had a better docking score against VEGFA (−7.4) and three amino acid residues with a strong hydrogen bond distance (Supplementary Table 3). Furthermore, ferulic acid demonstrated moderate docking scores against all the hub proteins. The two best hydrogen interactions were found with each hub protein except the IL6 gene. Among these, VEGFA showed a better binding score of −6.9 with the two hydrogen bond interactions and a better bond length (B: ASP323–2.315 Å and B: ILE418–2.970 Å) (Supplementary Fig. 3, Supplementary Table 2). Protocatechuic acid docked with MMP9 showed a better binding score of −7.1 and five different amino acid residues with hydrogen bond distances (Table 2). The docking results demonstrated that quercetin, isorhamnetin, Irisflorentin, ferulic acid, and protocatechuic acid are potential regulators of obesity-associated hub genes. The mechanisms through which ferulic acid inhibits α-amylase and α-glucosidase were investigated by Li et al.^37^. These five compounds revealed the highest docking scores and hydrogen bonding interactions out of 17 FBR bioactive metabolites.

### Quantification of network pharmacology and molecular docking-guided metabolites

**Fig. 3:**
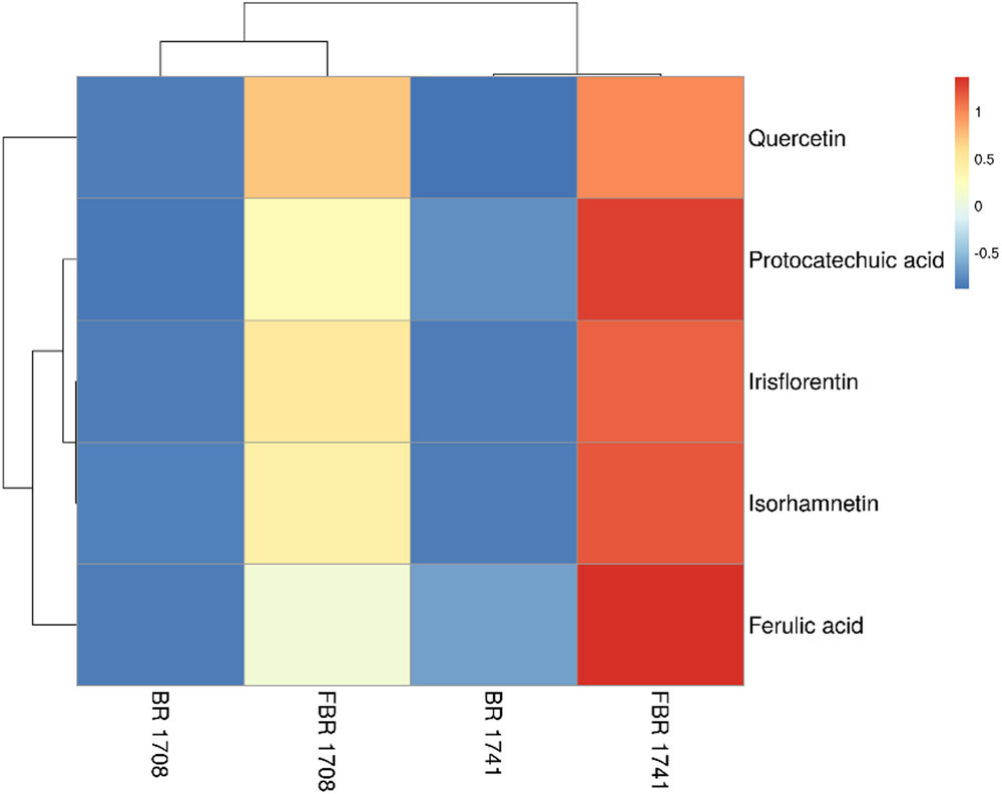
Quantification of polyphenolic metabolites in rice varieties. Heat map represent the levels of ultra high-performance liquid chromatography (UHPLC) polyphenolic metabolites in raw brown rice compared to fermented brown rice (FBR) varieties, FBR-1708 and FBR-1741; raw brown rice (BR) BR-1708 and BR-1741 at a concentration of 1 mg/mL.

In silico-determined metabolites were detected in the RBR and FBR (1708 and 1741) samples to assess the efficacy of fermentation on polyphenolic content (Fig. 3). Since ferulic acid has been shown to improve metabolic health in several ways, it was considered in this investigation. After 48 h of FBR-1741, ferulic acid was significantly higher than in the initial sample and other varieties (RBR-1741: 175.2 µg/g; FBR-1741: 570.2 µg/g). Doses for future investigations based on quantification and metabolites.

### In vivo *C. elegans* correlation of lifespan and lipid reduction mechanism by FBR extracts with metabolites

**Fig. 4:**
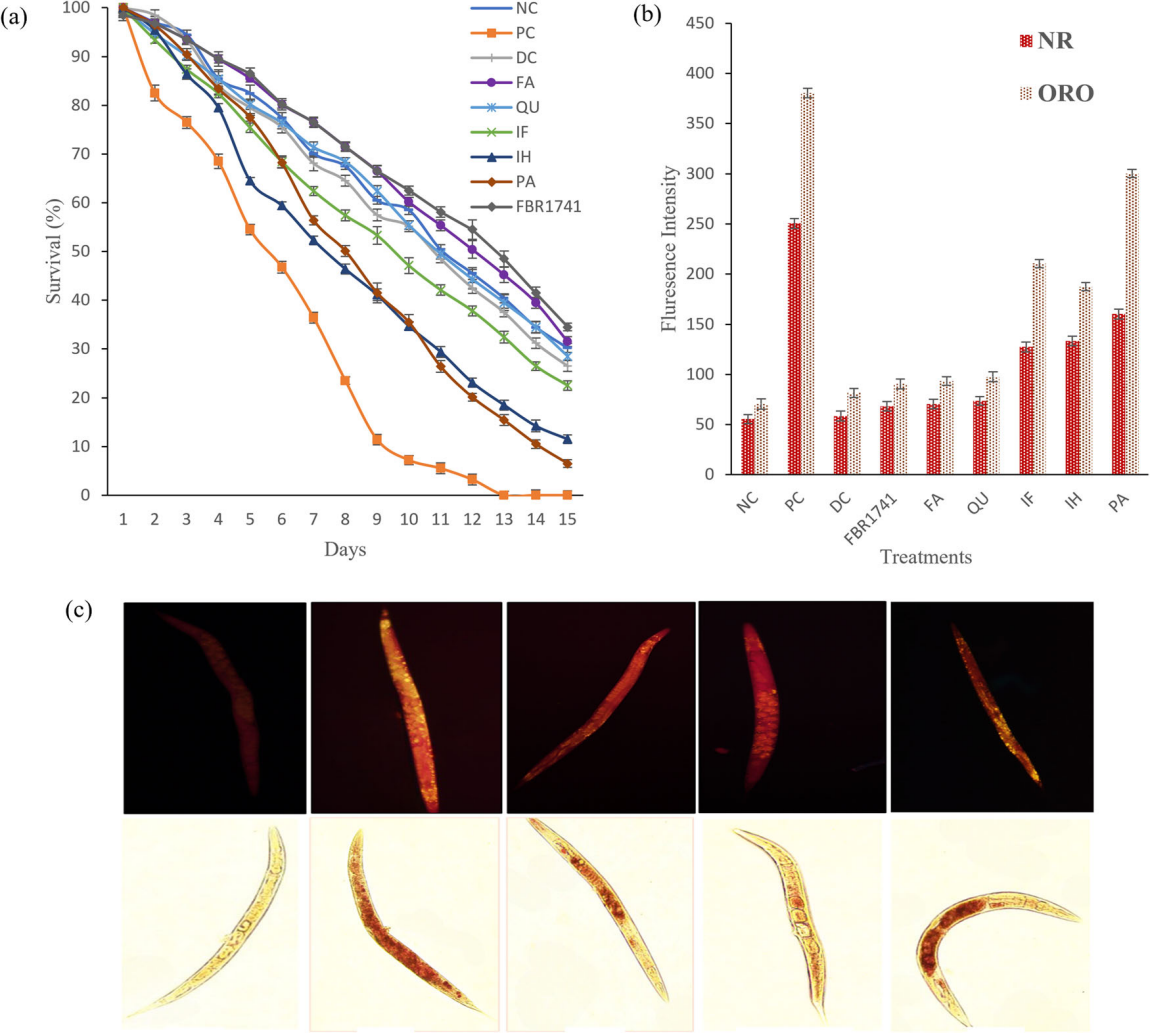
Impact of FBR-1741 on in vivo *C*. elegans model study. **a** Lifespan analysis. **b** *C. elegans* lipid droplets visualized by Nile-red staining and Oil Red. **c** mean of fluorescence intensity measured by Image J software; NC-OP50, PC-OP50 + Glucose, DC-OP50 + Glucose + Orlistat, FBR1741-OP50 + Glucose + FBR1741 (1 mg/mL), FA-OP50 + Glucose + FA, QU-OP50 + Glucose + QU, IF-OP50 + Glucose + IF, IH-OP50 + Glucose+IH, PA-OP50 + Glucose + PA. The data are shown as means SEM, with *p* < 0.05 indicating statistical significance. 10.6084/m9.Figureshare.24781494.

High-glucose diets may shorten the lifespan of *C. elegans*. The mean and maximum longevity (34.5 ± 1.5%) of the FBR-1741 groups were significantly longer than those of the other brown rice groups and those of the negative control groups (30.2 ± 1%). Based on network analysis, we evaluated the lifespan for the FBR-guided top five metabolites from network pharmacology, such as ferulic acid (31.5 ± 1.4), quercetin (28.5 ± 1), isorhamnetin (11.5 ± 09), irisflorentin (22.5 ± 0.8), and protocatechuic acid (6.5 ± 1). The lifespan was prolonged in the ferulic acid-supplemented diet group compared to the other metabolite groups. Ferulic acid, directly associated with a longer lifespan, was also elevated by FBR1741 (Fig. 4a). Obesity-induced N2 *C. elegans* consumed OP50 and glucose (PC) groups and died on the 13th day. Our results confirmed those of a previous investigation that suggested that FBR (mixed variety) might increase longevity following a high glucose diet^16^. Many fermented materials demonstrate that lipid metabolism regulates *C. elegans* longevity by connecting apoptosis, embryonic stem cells, and chromosomal factors. According to Hou et al.^38^, zymolytic grain extract increased longevity dose-dependently compared to the controls. This research shows that extracts from MNL5 FBR-1741 protect against the shorter lifespan caused by hyperglycemia by reducing fat accumulation.

### The effects of FBR extract on fat accumulation on *C. elegans* TG and FFA levels

**Fig. 5:**
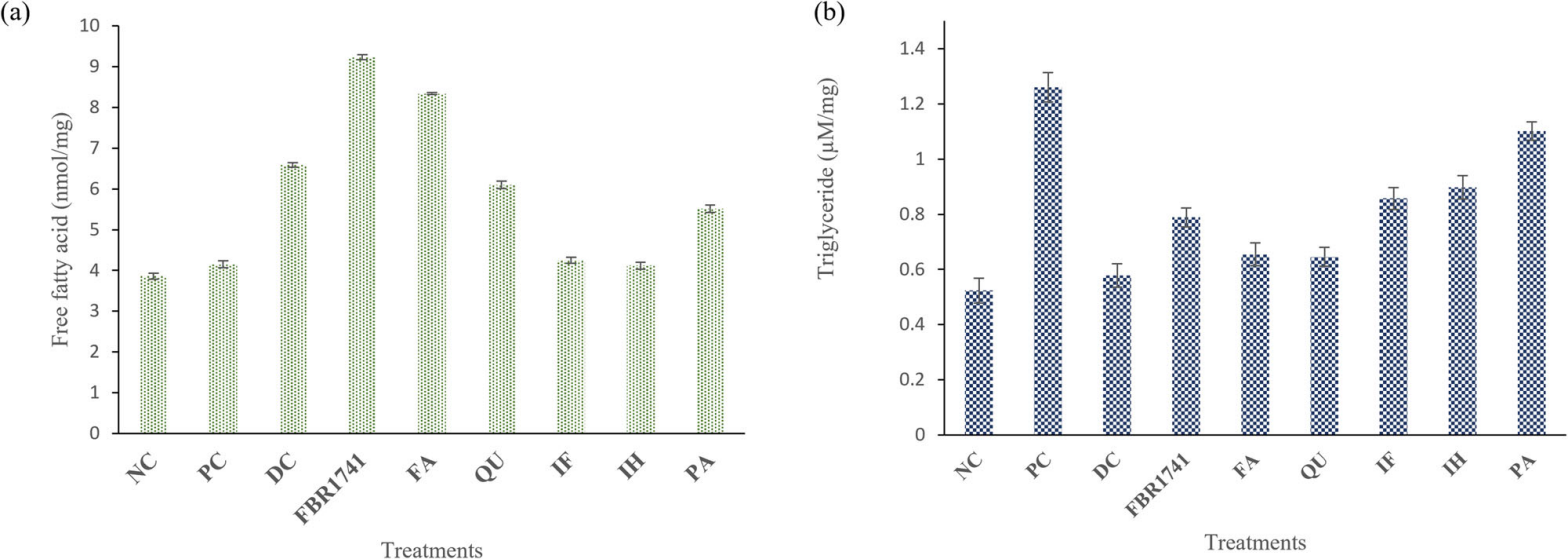
Effects of FBR-1741 and different metabolites treatment analysis. **a** Fatty acid levels. **b** Triglyceride level. The data are shown as means SEM, with *p* < 0.05 indicating statistical significance. 10.6084/m9.Figureshare.24781503.

Nile red fluorescence, TG, and FFA assays were used to determine the nematode fat deposition. Nile red fluorescent probe for intracellular lipids and proteins with hydrophobic domains (excitation/emission maxima ∼552/636 nm). Figure 4a shows that the worms in the treatment containing more fat ate more glucose than those in the NC treatment, implying that the worms’ diet was a major contributor to their increased fat content. Compared to the FBR-1741 and FA groups, less fat was found (Fig. 4a, b). Figure 4c demonstrates that FBR and FA inhibited TG in a dose-dependent manner, and the inhibitory effect was comparable to the Nile red and ORO staining findings. The genetic basis of fat metabolism has been extensively investigated using the *C. elegans* in vivo model. The metabolism, nutrient absorption, and reduction and deposition of fat were all carried out by the intestinal cells of *C. elegans*. TG enhancement is usually assessed as an endpoint when assessing food intake and energy expenditure. When administered to obese worms, FBR-1741 and FA increased their levels of free fatty acids. Normal, obese, FBR-174, FA, and orlistat worm groups had 3.86, 4.15, 9.23, and 6.59 nmol/mg free fatty acids, respectively (Fig. 5a). TG content increased in the obese model, then *C. elegans* treated with 10% glucose and FBR-1741 and FA decreased strongly (Fig. 5b). There are numerous possible explanations for this observation. The primary sources of free fatty acids are the breakdown of fat stores and dietary sources. Because of this, it is possible that an enhanced flux of free fatty acids in the worms is attributable to an abundance of these metabolites in the FBRs. Plant extract may also stimulate adipose enzymatic metabolism of endogenous fat, increasing plasma-free fatty acids. Compounds in plant extracts, in particular hormone-sensitive lipase, may catalyze the breakdown of adipose tissue triglycerides into fatty acids and glycerol. According to Rodrigues et al.^39^, specific phenolic compounds can promote lipolysis or the breakdown of triglycerides into glycerol and free fatty acids while inhibiting adipose growth and triglyceride production at the cellular level. Following Li et al.^37^, FA was analyzed with Oil Red O to investigate the significance of FA in suppressing fat accumulation. Furthermore, phenolic compounds can impact signaling pathways involved in adipogenesis. FBR-1741 can suppress lipid accumulation in *C. elegans* and extend its lifespan, according to moderate-to-strong connections between target lipid metabolism genes.

### Impact of FBR extracts on gene expression levels of *C. elegans*

There are many intricately controlled pathways involved in lipid metabolism in *C. elegans*. This study explored the levels of sterol regulatory element binding protein (*sbp-1*) and other important transcription factors that stimulate lipid production. In addition, we examined nuclear hormone receptor 49 (nhr-49), a transcription factor that promotes both fatty acid desaturation and β-oxidation, as a functional homolog of human peroxisome proliferator-activated receptors (PPARs)^40^. De novo lipogenesis-involved fatty acid desaturases (*fat-4, fat-5, fat-6*, and *fat-7*) are also positively regulated by nhr-49^41^. In addition, in *C. elegans*, *Daf-16* is a key gene transcription factor that controls the insulin/IGF-1 regulatory system and promotes survival. *Daf-16* is a gene primarily responsible for lifespan expansion, while it might also have an impact on fat accumulation in *C. elegans*^18^. Furthermore, *hosl-1* specifies the *C. elegans* homolog of hormone-sensitive lipase^42^, which triggers the primary rate-limiting process in triglyceride breakdown (Supplementary Fig. 4).

**Fig. 6:**
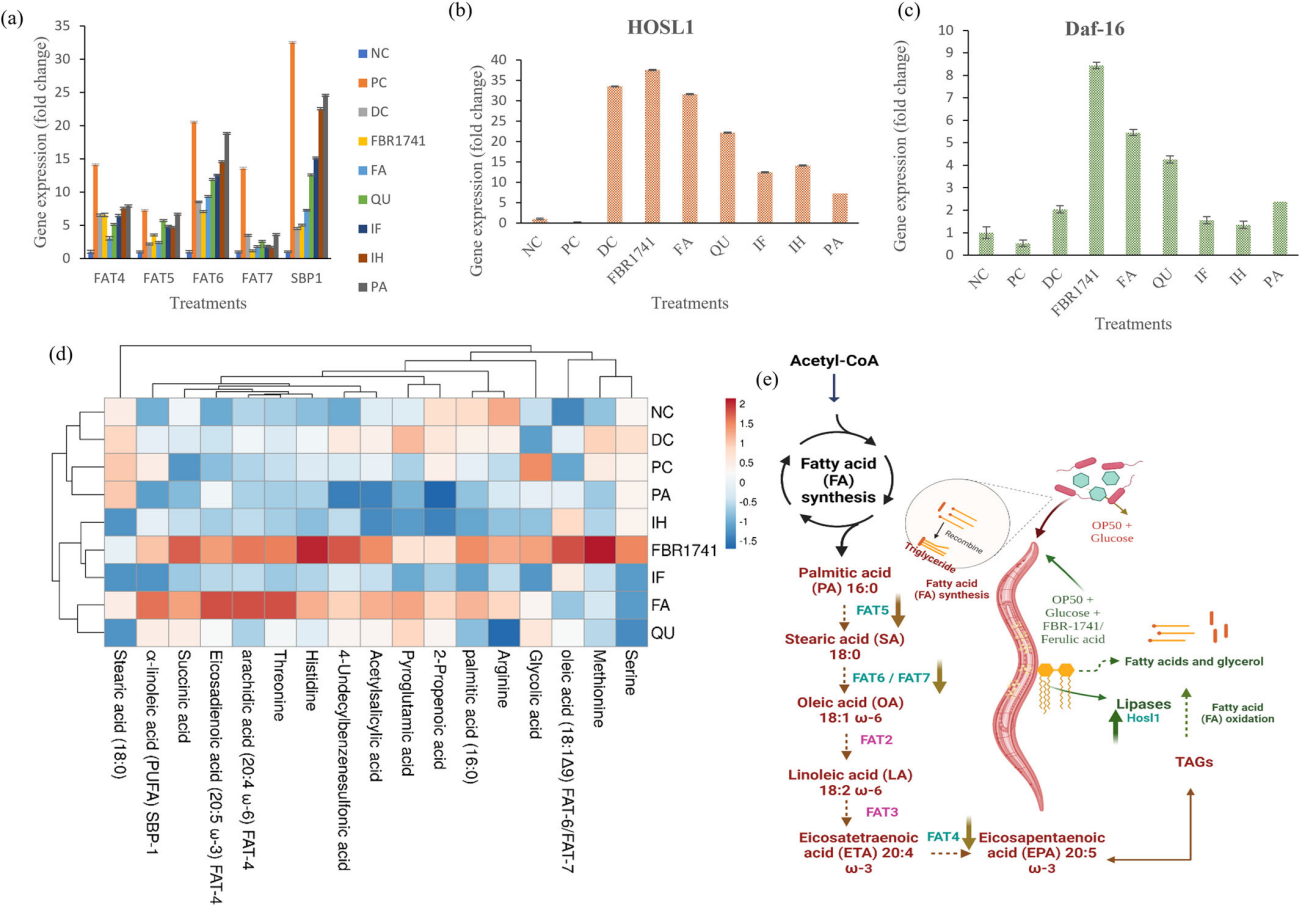
Gene expression profile on *C. elegans* impact of FBR-1741 and different metabolites. **a** Expression levels of genes involved in fat synthesis (FAT-4, FAT-6, FAT-7, and SBP-1). **b** Expression levels of genes involved in lipolysis (HOSL1). **c** Expression levels of genes involved in lifespan (Daf-16). **d** Heat map for the fatty acid metabolite profile. **e** Fat synthesis is responsible for fatty acid metabolites. The data are shown as means SEM, with *p* < 0.05 indicating statistical significance. 10.6084/m9.Figureshare.24781509.

**Fig. 7:**
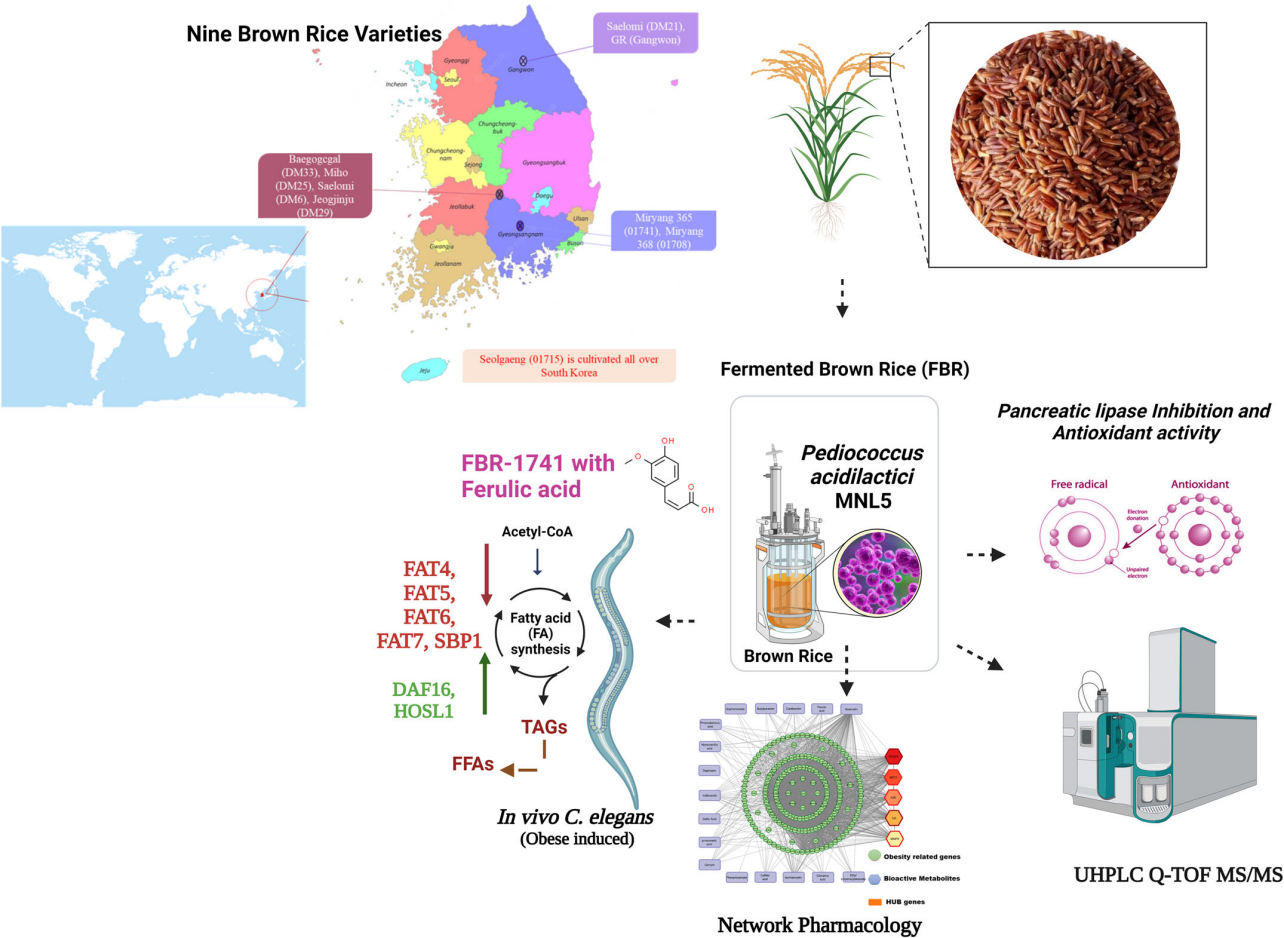
Impact of fermented brown rice on anti-obesity: a metabolomics approach. This study provides an in-depth analysis of untargeted metabolomics and network pharmacology, highlighting the potential of fermented brown rice in enhancing anti-obesity efficacy. Through comprehensive research, we explore the complex interactions and biochemical pathways influenced by fermented brown rice, offering new insights into its therapeutic potential.

Using qPCR, the FBR-1741 and metabolite samples were examined to determine how the fat-controlling genes in *C. elegans* had changed. As depicted in Figs. 6and 7, with the consumption of FBR-1741, the downregulation of *sbp-1*, *fat-4*, *fat-5*, *fat-6*, and *fat-7* genes and upregulation of hosl-1 and daf-16 by N2 model-related lipid accumulation, lipolysis, and lifespan were observed (Fig. 6a–c). In our study, Daf-16 activity in neurons enhanced life expectancy by above 20%. In this study, we discovered that *C. elegans*, like mammals, accumulate fat when they consume excess calories from sugar. The mechanism for fat accumulation in *C. elegans* is nearly identical to that of mammals, suggesting that this nematode could be a time-saving and cost-effective alternative for investigating fat metabolism.

### Effect of FBR extracts on fatty acid profiles of *C. elegans*

Human health is significantly affected by fatty acids, which consist of both saturated and unsaturated fatty acids. Saturated fatty acids are found primarily in palmitic, stearic, and arachidic acids; an excess of such acids can lead to atherosclerotic and cardiovascular disease^43^. The synthesis of unsaturated fatty acids depends on the genes that encode stearoyl-CoA desaturase (SCDs), such as fat-4, fat-5, fat-6, and fat-7. According to research, preventing the expression of fat-6 and fat-7, which encode the stearoyl CoA desaturating enzyme responsible for desaturating octadecyl saturated fatty acid, may minimize nematode fat accumulation^44^. Changes in metabolite levels occur in nearly all metabolic diseases. Several digestive enzymes that convert acetyl CoA to malonyl CoA and C16 palmitate regulate fat synthesis in *C. elegans*. This fluctuation may be restored to normal with the adoption of a nutritious daily diet (Supplementary Fig. 5a–c). Thus, this approach was used to show how metabolic alterations caused by obesity can be reversed by eating FBR-1741. Figure 6d shows that obese-induced FBR-1741 and FA treatment dramatically increased saturated fatty acid components, including palmitic acid (C16:0), stearic acid (C18:0), oleic acid (18:1), arachidic acid (20:4), eicosadienoic acid (20:5), and α-linoleic acid (PUFA) (Fig. 6d, Supplementary Fig. 5d, e). In addition, Qi et al.^45^ demonstrated that the ω-3 fatty acid α-linolenic acid (ALA) is able to increase the lifespan of *C. elegans*. Other treatments, such as positive control and metabolite diet groups, resulted in a significant decrease in saturated fatty acids. Serine, arginine, histidine, threonine, methionine, and glycolic acids also increased in FBR-1741- and FA-treated groups. According to Edwards et al.^46^, the levels of amino acids such as leucine, histidine, methionine, and tryptophan could significantly influence the lifespan of *C. elegans*. This finding provided further confirmation of our prior results, showing an increase in free fatty acid in worms after supplementation with FBR-1741, including metabolites for *C. elegans*. Results from both gene expression analysis and metabolomics assessment validate our prior finding that administering FBR-1741 and an FA-supplemented diet denoted that worm dramatically increased their lifespan (Supplementary Fig. 5f) and decreased lipid accumulation.

In this research, nine different types of brown rice were fermented with *P. acidilactici* MNL5 KCTC15156BP. Among the nine varieties, FBR-1741 had the strongest lipase inhibition, antioxidant activity, and phenolic phytochemical identification. In our previous investigation, we evaluated the biological activities of raw brown rice. In this study, we integrated omics data following systems biology principles to discover novel obesity therapeutic targets. However, FBR-1741 refers to specified phenolic compounds with potent lipase inhibition and antioxidant activity. Based on pathway enrichment studies, enhanced polyphenols may interfere with many pathways, including those implicated in obesity and obesity-related disorders, which may explain the possible anti-obesity impacts of FBR1741. The network-based pharmacological investigation of FBR-1741 found 17 compounds and 132 obesity-related target genes. Our results in the *C. elegans* N2 obese-induced paradigm reveal that FBR-1741 with ferulic acid has a higher survival rate and is a valuable indicator for assessing lipid reduction beyond dietary efforts. Ferulic acid was increased by FBR-1741, which may have a causal relationship with increased longevity and decreased lipid levels. In addition, FBR-1741 prolonged *C. elegans* lifespan and reduced fat accumulation, validating gene expression for fatty acid metabolites. However, additional research is needed to explain the fermented material for the in vivo *C. elegans* metabolomic study. This research provides a better comprehension of the synergistic effects of microorganisms. In addition, the findings of this study will be useful for future research using obesity models in mice to investigate the effect of FBR-1741 on gut microbiota and obesity correlation studies. In addition, we can use these considerations to develop a fermented brown rice product to combat obesity.

## Methods

### Procurement of chemicals and plant materials

Experimental medium and chemicals were purchased from Daejung Chemicals and Metals Co., Ltd, Korea. A PicoSens^TM^ Triglyceride and Free Fatty Acid Assay Kit was purchased from BIOMAX in Seoul, Korea. Our study used METLIN and the Metabolomics Workbench to assist us in determining the classification of polyphenolics. Brown rice was collected from the Rural Development Administration (RDA) of the Republic of Korea. After the samples were powdered with an electric grinder, the materials were filtered using a sieve size of 40 mm to eliminate dust and debris^47^.

### Bacterial growth and optimization of brown rice fermentation

The brown rice fermentation medium was mixed on sterilized rice powder with distilled water (1:6 ratio). The fermentation of brown rice was autoclaved for 15 min at 121 °C before lactic acid bacteria were inoculated. The *P. acidilactici* MNL5 (KCTC15156BP) strain (2 × 10^7^ CFU/mL) was transferred and grown for 48 h at 37 °C with agitation at 200 rpm. The samples were freeze-dried and kept at −20 °C for further investigation.

### Solvent extraction method

Our study used METLIN and the Metabolomics Workbench to assist us in determining the classification of polyphenolics. A total of 50 g of fermented rice powder was placed in an electric shaker and mixed with 100 mL of 70% ethanol (1:20 w/v) at ~50 °C for ~4 h. The extracts were centrifuged at 4000×*g* for 10–15 min (Hanil Science Industrial, Incheon, Korea). This process was repeated three times. The supernatant’s ethanol content was evaporated at 50–55 °C and freeze-dried. Then, the samples were stored at −20 °C. The samples were prepared into a standard liquid sample with 1 mg/mL of concentration^47^.

### In vitro anti-obesity efficacy by using pancreatic lipase inhibition assay

The lipase inhibition assay of the FBR samples was performed in a 36-well plate with minor modifications^16^. The percentage of inhibition of lipase was used to express the results. Lipase inhibition with and without substrate was determined using fluorescence readings. The results are provided as a percentage of inhibition of 1 mg/mL of the samples.

### Total phenolic content (TPC) and total flavonoid content (TFC)

The FBR samples were evaluated for TPC and TFC utilizing a 36-well plate, following the methodology described by Glorybai et al.^48^. For the TPC, 200 µL Folin–Ciocalteu reagent was added to 100 µL sample extracts, standard, or 95% (v/v) methanol as blank. Following vertexing, the mixture was incubated at an ambient temperature for 2 h. After adding 800 µL of 700 mM sodium carbonate to each mixture, absorbance was measured at 765 nm.

For the TFC, 250 µL sample extracts were pipetted into microplate wells, followed by 75 µL N_a_NO_2_ (50 g L^−1^) and 1 mL distilled water. After 5 min of settling, 75 µL AlCl_3_ (100 g L^−1^) was added. A total of 500 µL of 1 M NaOH and 600 µL distilled water were added and incubated for 6 min after letting the reaction mixture settle. After 30 s of shaking, a Spectra-Max i3 plate reader (Molecular Devices Korea, LLC, Seoul, Korea) measured absorbance at 510 nm. TPC and TFC results were reported in gallic acid and catechin equivalent per 100 g (mg/100 g, DW).

### DPPH, ABTS, and FRAP radical scavenging effects

The FBR sample was examined with DPPH, ABTS, and FRAP on a 36-well plate using the previously described process with minimal changes^49^.

In brief, 100 μL of sample extract, standard (Trolox), or blank (methanol) was mixed with a freshly prepared 500 μM DPPH solution in a 24-well microplate. Then, this was incubated at room temperature for 30 min. Absorbance was measured at 515 nm. The baseline curve was the Trolox concentration plot with DPPH radical scavenging activity.

ABTS stock solution was prepared by mixing 2.45 mmol/L potassium persulfate with 7 mmol/L ABTS solution (1:1, v/v) in the dark for 12–16 h at room temperature. The ABTS + reagent was diluted with methanol until it achieved 0.700 ± 0.020 absorbance at 734 nm wavelength. After diluting 100 μL of extracts or standards with 1 mL of ABTS + solution, absorbance was measured at 734 nm.

To prepare the FRAP reagent, 0.1 mL of extract was diluted with 3.9 mL of acetate buffer (50 mL, 0.3 M, pH 3.6), TPTZ solution (10 mM in 40 mM HCl), and FeCl_3_·6H_2_O (5 mL, 20 mM) for 10 min at 37 °C. Absorbance was measured at 593 nm wavelength. The absorbance was measured with the SpectraMax i3 plate reader (Molecular Devices Korea, LLC). DPPH, ABTS, and FRAP values were expressed as mg Trolox equivalent per 100 g of sample (mg Trolox equiv./100 g, DW) using the following formula:

$${\rm{Radical}}\; {\rm{scavenging}}\; {\rm{activity}}( \% )=({\rm{Ac}}-{\rm{Ae}})/{\rm{Ac}}\times 100$$

where Ae represents the extract or standard absorbance; Ac represents the blank sample absorbance.

### Identification and evaluation of untargeted metabolites in FBR varieties using UHPLC-Q-TOF-MS/MS

Characterizing untargeted metabolites using the UHPLC-Q-TOF-MS/MS method: The filtered samples were added to an autosampler vial for analysis using 0.22 mm Micropore injection filters from Merck KGaA, Darmstadt, Germany, following our prior studies^16^. After setting the Q-TOF-MS/MS to the negative mode, the mass range was set to 100–1000, and the precision was set to 5000. For full mass spectra, 1.45 kV and 30 V capillary and cone voltages were utilized. N_2_ flowed at 900 L/h, while helium (the cone gas) flowed at 45 L/h. MS/MS spectra were obtained at 15, 20, and 30 V collision energies with N_2_ at 250 °C and the ion source at 120 °C. Untargeted metabolites were found through a comparison of retention time (RT) along with spectral profile spectral databases, such as XCMS Online (METLIN; https://metlin.scripps.edu) and the Metabolomics Workbench (https://www.metabolomic-micsworkbench.org).

### Correlation and identification of bioactive metabolites by network pharmacology in fermented brown rice (FBR) for anti-obesity

The bioactive components in FBR were identified by employing UHPLC-Q-TOF-MS/MS. The resultant metabolites were subsequently assessed by the Traditional Chinese Medicine Systems Pharmacology (TCMSP) database for assimilatory characteristics according to drug-selection criteria such as oral bioavailability (OB) and drug-likeness (DL). Oral bioavailability summarizes medicines’ attributes of absorption, distribution, metabolism, and excretion (ADME) and their consumption in the blood flow^50^. The chemical data for each ingredient, including their chemical structures, molecular formula, and biological information, were acquired using the Pub Chem and Drug Bank databases.

### Obesity-related gene target screening

Obesity-related gene target data were obtained from TCMSP, DisGeNET, and GeneCards^50^. In addition, the aforementioned electronic databases were explored using the search term “obesity” to find the disease of interest and its associated genes.

### Functional-enrichment analysis

The network of protein‒protein interactions (PPI) was constructed using STRING 11.0 and was estimated toward a high confidence level of 70% identity (0.700). The enrichment analyses, Gene Ontology (GO), and KEGG Pathway were performed to develop the biological process (BP), molecular function (MF), and cellular components (CC) of disease target proteins^50,51^. Moreover, networks of hub-gene-associated targets, pathway interactions, and target‒gene interactions with potential compounds were constructed using the Cystoscope tool^50^.

### In silico molecular docking

The tertiary molecular structures of the top-hit hub genes and secondary metabolic products were obtained from the Drug Bank (a pharmaceutical database) and Data Bank (https://www.rcsb.org). Eliminating hydrogen from the receptor and omitting water molecules generated protein molecular structures. Flexible docking was used to evaluate molecular interactions between top-hit hub proteins and ligands using AUTODOCK VINA, and binding pockets were autonomously identified. The Discovery Studio software was used to determine the molecular binding from a two-and three-dimensional viewpoint. The docking scores, bond lengths, and all interactions were recorded^50^.

### Impact of the FBR-supplemented *C. elegans* model on the lipid reduction mechanism

*Caenorhabditis elegans* (N_2_ wild type) and *Escherichia coli* OP50 were acquired from the Caenorhabditis Genetics Center (CGC), University of Minnesota, Minneapolis. The growth, synchronization, and maintenance of *C. elegans* took place at 20 °C. Nematode growth medium (NGM) plates contain 5-fluorodeoxyuridine (FUDR, 140 mM) and were inoculated at 37 °C with 10% glucose with FBR-1741 (1 mg/plate) and a different metabolite diet. After 4 h, worms were moved to a fresh NGM 90 mm Petri dish with a targeted metabolite diet^52^. The dosage was used guided by UHPLC quantification of FBR-1741 (1 mg/ml): quercetin (0.19 µg/plate), isorhamnetin (0.18 µg/plate), irisflorentin (0.18 µg/plate), ferulic acid (0.57 µg/plate), and protocatechuic acid (0.19 µg/plate).

### Impact of FBR and bioactive metabolites on the *C. elegans* lifespan

FBR-1741 samples were evaluated by our previous procedure, and *C. elegans* longevity was evaluated^16^. The nematode was maintained, developed, and synchronized with bleached gravid hermaphrodites (1:1 ratio of NaOH and sodium hypochlorite). In the bleach solution added, vortexed with eggs, and centrifuged at 1500 rpm for 2 min. After adding an equivalent buffer volume, M9 buffer was used to wash the egg pellet (3 g KH_2_PO_4_, 6 g Na_2_HPO_4_, 5 g NaCl, 1 mL 1 M MgSO_4_, H_2_O to 1 L). The L1-stage worms were transferred to plates of nematode growth medium (NGM) containing *E. coli* OP50 to be fed to the worms at a temperature of 20 °C. This study incubated 50 μL of OP50 in LB broth on a 60 mm NGM Petri dish (FUdR) at 37 °C for 24 h. FBR, a diet with specific metabolites, and OP50 plates were given to 50 L4-stage *C. elegans*. Each plate was placed in an incubator at 20 °C, and every 24 h, dead worms were enumerated. Worms were moved to new NGM plates containing the specified diet every three days. To determine the effect of glucose on FBR, the average lifespan of OP50 nematodes was evaluated.

### Lipid accumulation in *C. elegans*: a fluorescence microscopy study

Nile red and Oil Red dyes were used for the lipid reduction experiments. In this study, L4-stage worms were rinsed with M9 buffer. Furthermore, after adding a drop of Nile red (0.05 μg/mL) solution, the worms were incubated for 30 min and rinsed twice with 25% ethanol. In addition, 60% isopropanol was added for 5 min, and L4 worms were collected in 15 mL centrifuge tubes. The worm pellet was transferred confocal dish under the fluorescence microscope (Olympus CKX53, Tokyo, Japan)^52^. Furthermore, 0.05 μg/mL of Oil Red O solution was used in accordance with the above procedure. Individual worms were photographed using an Olympus SZ 61 zoom stereomicroscope and an HK3.1 CMOS camera on worm pellets to be treated with ORO and subsequently transferred and analyzed. Lipids stained were predominantly present in the intestine, and the anterior region was much brighter than the posterior region. The area used for integrating fluorescence density measurements was chosen from the intestinal anterior front to the vulva. Six L4 worms were randomized for analysis. For the image J analysis, the images were captured at ×20 magnification.

### Determination of triglycerides and free fatty acids

Triglyceride (TG) and free fatty acids (FFA) analysis measures the effects on lipid levels of different FBRs with metabolites in mixed diets in *C. elegans*. TG and FFA levels were measured by measuring *C. elegans* at 50% of the lifespan for treatments. Triglycerides and FFA content were determined using a commercially available colorimetric assay kit (Biomax, Seoul, Korea), following the manufacturer’s guidelines.

### Gene expression profile of the FBR-1741 and metabolite-supplemented *C. elegans* model

Worms were collected from a 90 mm Petri dish on the treatment plate at 20 °C on the 7th day of 50% lifespan. Each treatment collected 1000 worms, washed with M9 buffer, and centrifuged at 5000 rpm for 5 min. The worm pellet was added, together with TRIzol reagent (TRIzol® Thermo Fisher Scientific, Inc., Middletown, VA), followed by lysing in 2 mL tubes with Lysing Matrix Z beads (MP Biomedicals, Santa Ana, CA, USA) was used to extract total RNA from worm samples. Following the manufacturer’s instructions, a high-capacity cDNA reverse transcription kit (Thermo Fisher Scientific, Inc., Middletown, VA) and a standard thermal cycler (Bio-Rad Laboratories Inc., Hercules, CA) produced cDNA templates. The 260/280 absorbance ratio determined the RNA purity. StepOnePlus Real-Time PCR (Applied Biosystems, Foster City, CA) and GoTaq® qPCR Master Mix kit (Promega) were used to measure gene expression. Each reaction contained 10 μL of GoTaq® qPCR Master Mix kit (A600A), 2 μL of cDNA (10 ng μL^−1^), 0.4 μL of each primer (10 pg μL^−1^), and 7 μL of double-distilled water. The 2^−^^∆∆^CT technique standardized gene expression levels to the act-1 primer (Supplementary Table 4).

### Worm sample preparation for metabolomics analysis

Approximately 1000 worms were collected for each treatment, washed in M9 buffer, and centrifuged at 5000 rpm for 5 min. The worm pellet was added to a Lysing Matrix Z (MP Biomedicals, Bio-Connect, The Netherlands) vial containing 1 mL of 70% methanol, and the mixture was homogenized. The previously mentioned metabolomic analysis technique was carried out on the supernatant collected following centrifuging the samples for 10 min at 10,000 rpm and filtering through a 0.45 m membrane filter.

### Statistical analysis

All evaluations were carried out at least three times, and the results are expressed as the mean ± SD. Microsoft Excel 365 enabled the acquisition of pertinent graphs. The tertiary structures of the top hit hub proteins and derived chemical compounds were retrieved from the protein data bank (https://www.rcsb.org/) and DrugBank (a familiarity foot of the pharmaceutical repository) (https://go.drugbank.com/). The protein structures were prepared based on the addition of hydrogen molecules and the removal of water molecules in the receptor. The molecular docking studies were performed with the selected top-hit hub proteins and ligands using the AutoDock Vina 4.2.6. The empirical formula was applied to find chemicals from PubChem (accessed on March 12, 2022) and ChemSpider (accessed on March 20, 2022). Heatmaps were generated by the ClustVis programs (https://biit.cs.ut.ee/clustvis/accessed on May 16, 2023)^16^.

### Reporting summary

Further information on research design is available in the Nature Research Reporting Summary linked to this article.

### Supplementary information

Supplementary Tables and Figures

Reporting Summary

## Data Availability

We declare that all data related to this study are included in this paper and its supplementary information.
