# Supplementary material for: Untargeted metabolomics-based network pharmacology reveals fermented brown rice towards anti-obesity efficacy
Source: NPJ Sci Food. 2024 Mar 30;8:20. doi: 10.1038/s41538-024-00258-x (PMC10981755; doi:10.1038/s41538-024-00258-x)
Supplement: Supplementary file 1 — Supplementary Tables and Figures [file 41538_2024_258_MOESM1_ESM.pdf]

Supplementary Tables and Figures for

Untargeted Metabolomics-Based Network Pharmacology Reveals Fermented Brown Rice Towards Anti-Obesity Efficacy

Kaliyan Barathikannan [1,3], Ramachandran Chelliah [2,7], Annadurai Vinothkanna [4,5], Ragothaman Prathiviraj [6], Akanksha Tyagi [2], Selvakumar Vijayalakshmi [2], Min-Jin Lim [2], Ai-Qun Jia [5], Deog-Hwan Oh [2*.]

1. Agricultural and Life Science Research Institute, Kangwon National University, Chuncheon 24341, Korea.

2. Department of Food Science and Biotechnology, College of Agriculture and Life Sciences, Kangwon National University, Chuncheon 200-701, South Korea.

3. Saveetha School of Engineering, Saveetha (SIMATS) University, Tamil Nadu, 600124, India.

4. School of Life Sciences, Hainan University, Haikou, 570228, China.

5. Hainan General Hospital, Hainan Affiliated Hospital of Hainan Medical University, Haikou 570311, China.

6. Department of Microbiology, Pondicherry University, Puducherry 605014, India.

7. Kangwon Institute of Inclusive Technology (KIIT), Kangwon National University, Chuncheon 24341, South Korea.

* Corresponding author

Supplementary Table 1. Metabolites Identified in FBR 1708 by UHPLC-Q-TOF-MS^2. RT: Retention time.

S.No.	RT (min)	Tentative Compounds	Molecular Formula	Precursor Mass	Found at mass	Area	Adduct/ charge
1.	16.2	Ferulic acid	$C_{10}H_{10}O_4$	194.2800	194.0511	4.90E+04	[M-H]-
2.	6.44	4-Hydroxybenzoic acid	$C_7H_6O_3$	138.03246	137.0249	2.00E+05	[M-H]-
3.	14.16	Cinnamic acid	$C_9H_8O_2$	148.05308	147.0457	3.30E+06	[M-H]-
4.	11.7	Protocatechuic acid	$C_7H_6O_4$	154.02694	153.0198	9.90E+05	[M-H]-
5.	6.17	p-Coumaric acid	$C_9H_8O_3$	164.04775	163.0406	4.80E+04	[M-H]-
6.	14.16	Ethyl 4-hydroxybenzoate	$C_9H_{10}O_3$	166.06345	165.0559	8.10E+06	[M-H]-
7.	3.27	Norcantharidin	$C_8H_8O_4$	168.04246	167.0351	3.40E+06	[M-H]-
8.	10.59	Caffeic acid	$C_9H_8O_4$	180.04288	179.0354	7.00E+04	[M-H]-
9.	6.18	Homovanillic acid	$C_9H_{10}O_4$	182.05830	181.0511	3.20E+06	[M-H]-
10.	20.01	Butylparaben	$C_{11}H_{14}O_3$	194.09510	193.0874	3.50E+05	[M-H]-
11.	4.98	L-Tryptophan	$C_{11}H_{12}N_2O_2$	204.09051	203.083	7.70E+02	[M-H]-
12.	18.84	Quercetin	$C_{15}H_{10}O_7$	302.04249	301.0352	2.70E+06	[M-H]-
13.	19.65	Isorhamnetin	$C_{16}H_{12}O_7$	316.05840	315.0513	1.10E+06	[M-H]-
14.	17.19	Sophoricoside	$C_{21}H_{20}O_{10}$	432.10629	431.099	1.90E+05	[M-H]-

Supplementary Table 2. Representation of molecular properties from traditional Chinese medicine systems pharmacology database and analysis platform for the Metabolites Identified in MNL5 fermented brown rice (1741).

S.No.	RT (min)	Tentative Compounds	Molecular Formula	OB%	BBB	DL	HL
1.	16.12	Ferulic acid	$C_{10}H_{10}O_4$	39.56	-0.03	0.06	2.38
2.	14.16	Cinnamic acid	$C_9H_8O_2$	19.68	0.96	0.03	-
3.	11.7	Protocatechuic acid	$C_7H_6O_4$	25.37	-0.17	0.04	-
4.	6.17	p-Coumaric acid	$C_9H_8O_3$	43.29	0.13	0.04	4.43
5.	14.16	Ethyl 4-hydroxybenzoate	$C_9H_{10}O_3$	64.98	0.68	0.04	4.50
6.	10.59	Caffeic acid	$C_9H_8O_4$	54.97	0.11	0.05	1.63
7.	6.18	Homovanillic acid	$C_9H_{10}O_4$	35.47	0.09	0.04	11.62
8.	20.01	Butylparaben	$C_{11}H_{14}O_3$	64.98	0.68	0.04	4.50
9.	4.98	Gallic Acid	$C_{11}H_{12}N_2O_2$	31.69	-0.54	0.04	11.78
10.	18.84	Quercetin	$C_{15}H_{10}O_7$	46.43	-0.77	0.28	14.40
11.	19.65	Isorhamnetin	$C_{16}H_{12}O_7$	49.60	-0.54	0.31	14.34
12.	17.19	Sophoricoside	$C_{21}H_{20}O_{10}$	10.42	-2.22	0.78	-
13.	3.44	Phenprobamate	$C_9H_{11}NO_2$	2.42	0.94	0.04	-
14.	16.43	Daphnetin	$C_9H_6O_4$	24.23	0.28	0.07	-
15.	15.00	Cantharidin	$C_{10}H_{12}O_4$	51.23	0.15	0.10	13.50
16.	15.77	Genipin	$C_{11}H_{14}O_5$	26.06	-0.98	0.10	-
17.	6.19	Irisflorentin	$C_{20}H_{18}O_8$	12.52	-0.04	0.64	-

Supplementary Table 3. Molecular docking analysis of disease-associated hub genes against the potential FBR-1741 metabolites.

Receptor	Ligands	Binging Score	Hydrogen bond interaction	
			Amino acid Residues	Distance A°
VEGFA	Ferulic acid	-6.9	B:ASP323<br>B:ILE418	2.315<br>2.970
	Quercetin	-8.4	B:ASP323<br>B:ASP301	2.242<br>2.507
	Isorhamnetin	-8.5	C:TYR357<br>C:HIS415<br>B:TYR299<br>B:ILE418	2.243<br>2.059<br>2.918<br>2.406
	Protocatechuic acid	-6.4	C:TRP304<br>C:ASP323	2.622<br>2.991
	Irisflorentin	-7.4	B:ASN380<br>D:GLN353<br>D:ARG421	1.911<br>2.324<br>2.222
AKT	Ferulic acid	-4.9	A:ARG15<br>A:THR87	2.127<br>2.409
	Quercetin	-6.8	A:ARG86<br>A:THR87	2.429<br>2.134
	Isorhamnetin	-6.5	A:ARG86<br>A:GLU17	2.410<br>2.482
	Protocatechuic acid	-4.7	A:ARG41	2.316
	Irisflorentin	-5.8	A:ARG15<br>A:ARG86	2.326<br>2.443
JUN	Ferulic acid	-4.5	B:ARG9<br>B:ARG15	2.369<br>2.175
	Quercetin	-5.2	B:SER19<br>B:LEU26	2.306<br>2.978
	Isorhamnetin	-5.3	B:SER19	2.418
	Protocatechuic acid	-4.4	B:ARG11<br>B:ARG15<br>B:GLU8<br>B:GLU8	2.419<br>2.053<br>1.940<br>2.372
	Irisflorentin	-5.1	B:ARG15<br>B:ARG22	1.872<br>2.371
IL6	Ferulic acid	-5.6	-	-
	Quercetin	-7.2	A:ARG169	2.544

			A:MET68<br>A:GLU173	2.304<br>2.758
	Isorhamnetin	-7.1	A:ARG169<br>A:MET68<br>A:LEU63<br>A:ASN62	2.496<br>2.550<br>2.169<br>2.657
	Protocatechuic acid	-5.1	A:ARG105<br>A:ARG105<br>A:SER48	2.067<br>2.605<br>2.445
	Irisflorentin	-6.3	A:LYS67<br>A:MET68<br>A:MET68<br>A:SER170	2.243<br>2.258<br>2.321<br>2.174
MMP9	Ferulic acid	-7.0	A:PRO421	1.873
	Quercetin	-8.0	A:HIS405	2.300
	Isorhamnetin	-7.9	A:ARG51	3.007
	Protocatechuic acid	-7.1	A:THR426<br>A:ARG424<br>A:LEU418<br>A:TYR420<br>A:TYR420	2.963<br>1.855<br>2.728<br>2.086<br>2.340
	Irisflorentin	-7.2	-	-

Supplementary Table 4. *Caenorhabditis elegans* primers for detection of gene expression

Gene name	Forward primer	Reverse primer
**Fat-4**	TGGAGGTTTCCTGCTCTCTCA	TGGTAAACCATTTGCTGCTGC
**Fat-5**	CAACTACCATCACACCTTCC	CCCGTTCAGTTTCACAGCC
**Fat-6**	CAACTTCCATCACACATTCCC	TCCTCGTTGAATATCACATCC
**Fat-7**	TTTCCACCACACATTCCCAC	TCTTCACTTCCGTGATTGGC
**Spb-1**	GGCGGCGAAGATTGTGATTC	CGCTCGGTTTTTGGTCTTCG
**Daf-16**	CCAGACGGAAGGCTTAAACT	ATTCGCATGAAACGAGAATG
**Hosl-1**	DNAAAAGGCAACTTC-AGGACCACT	DNAGTCCGAACACATCTGTACCAAC

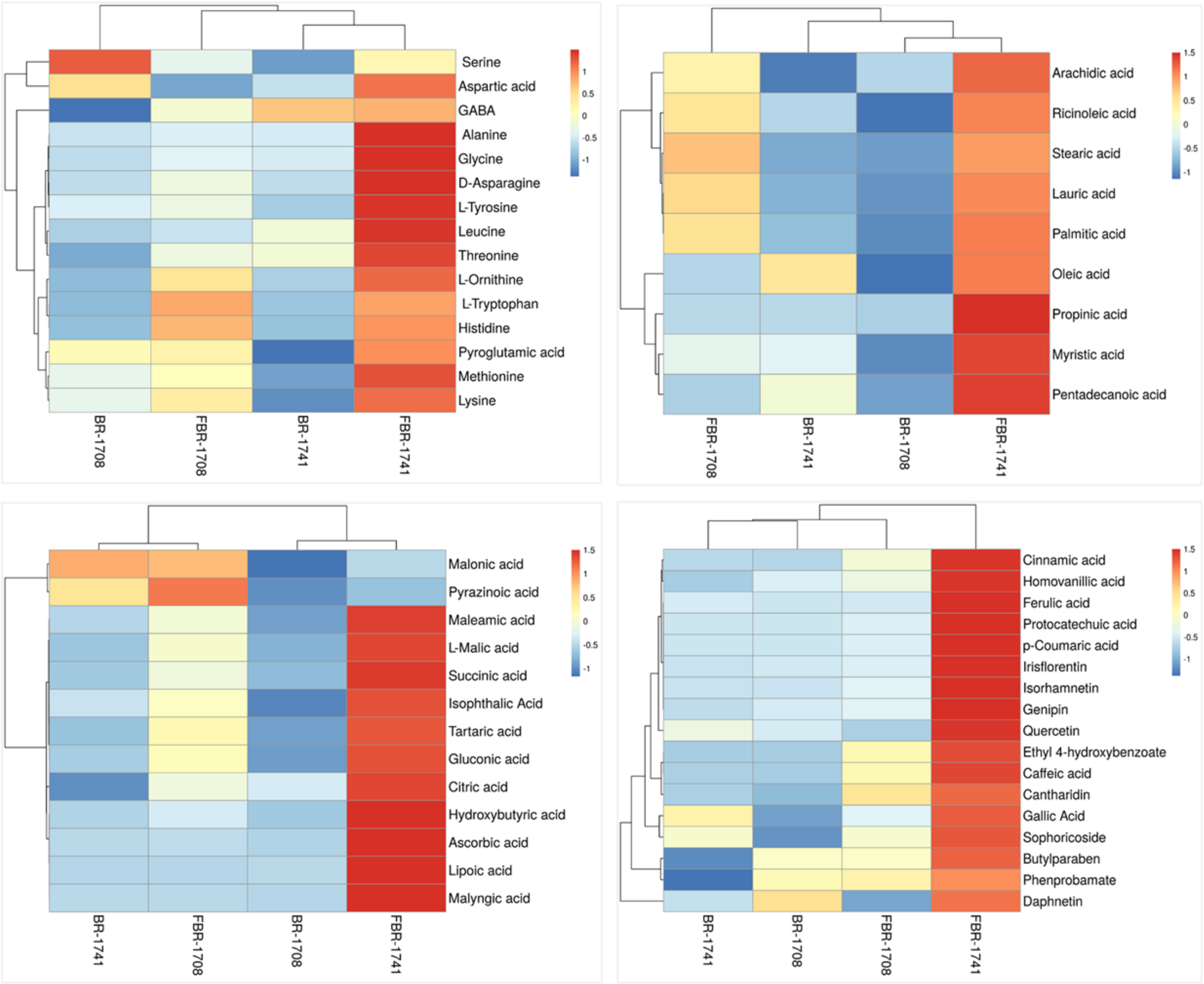

Supplementary Figure 1. Heat map plot showing the profile of Raw BR and FBR samples (1708 & 1741) (a) Levels of Amino acids; (b) Levels of Fatty acids; (c) Levels of Organic Acids; (d) Levels of phenolic compounds.

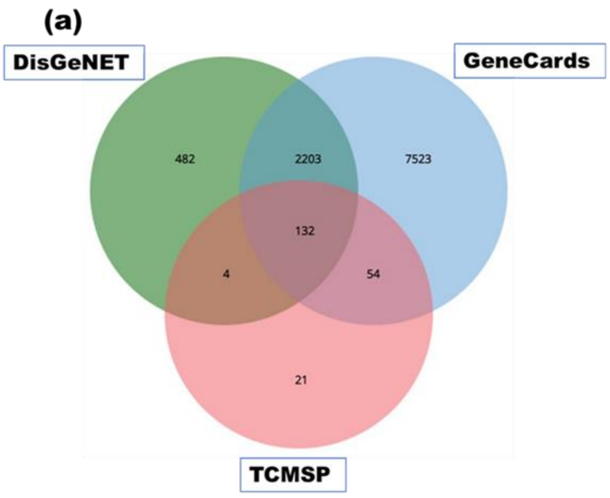

(a)

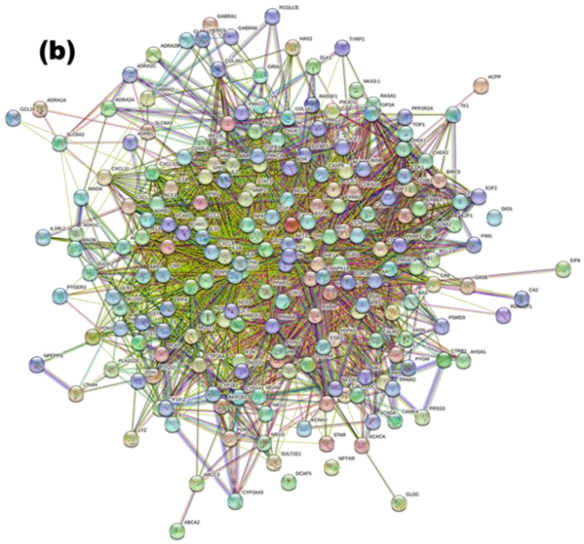

(b)

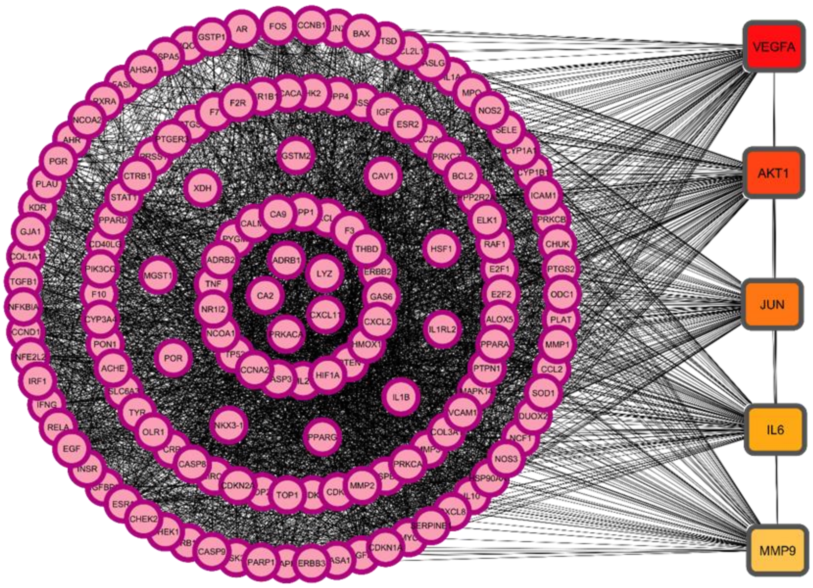

(c)

Supplementary Figure 2. (a) Venn diagram for comparing target receptors (132 genes) with fermented brown rice ingredients retrieved from TCMSP. DisGeNET and GeneCards databases: Genes associated with obesity-related diseases; (b) The enriched protein–protein interaction network analysis of genes encoding target receptors of obesity-related diseases. The enrichment of p-value is $p < 1.0e{-16}$; (c) Identification of top ten hub genes from the obesity-related disease genes interaction networks.

5DN2_VEGF-A VS 170569

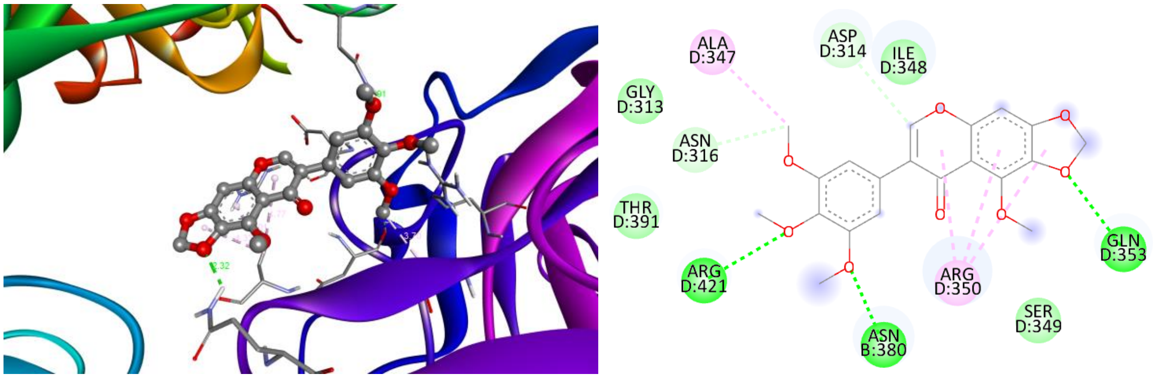

5DN2_VEGF-A VS 445858

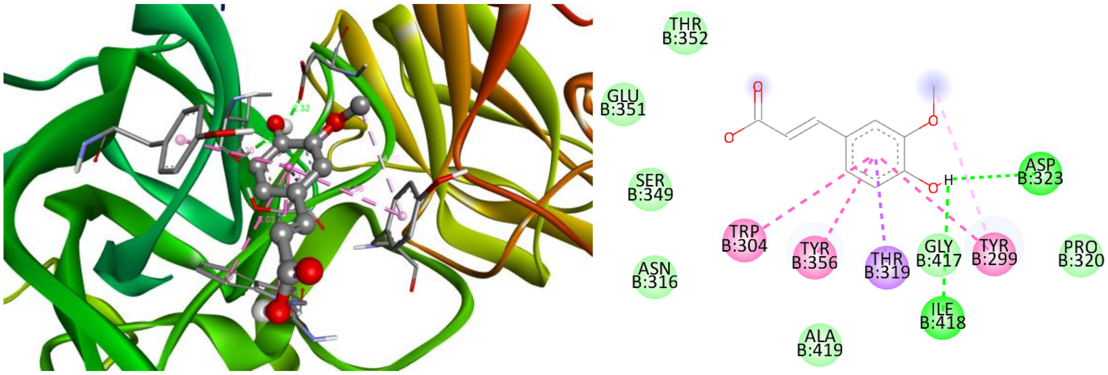

5DN2_VEGF-A VS 5280343

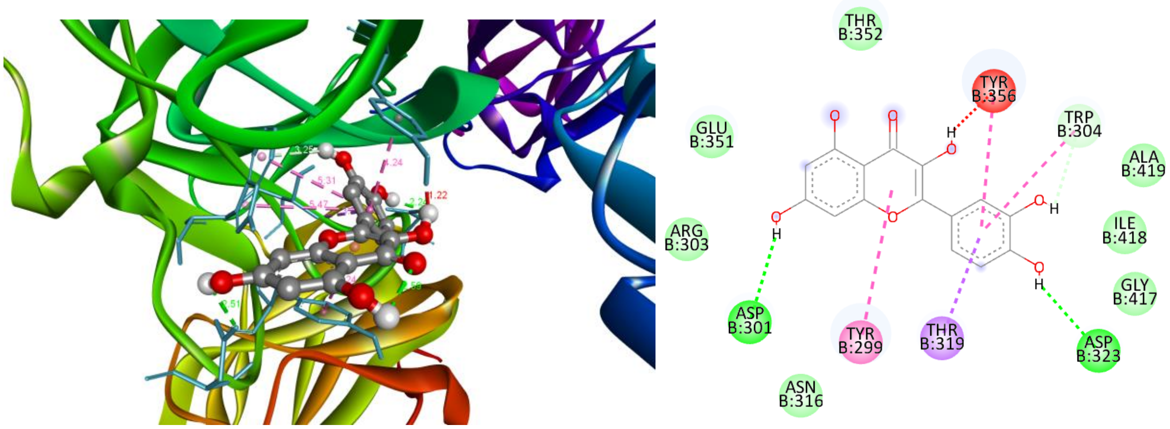

5DN2_VEGF-A VS 5281654

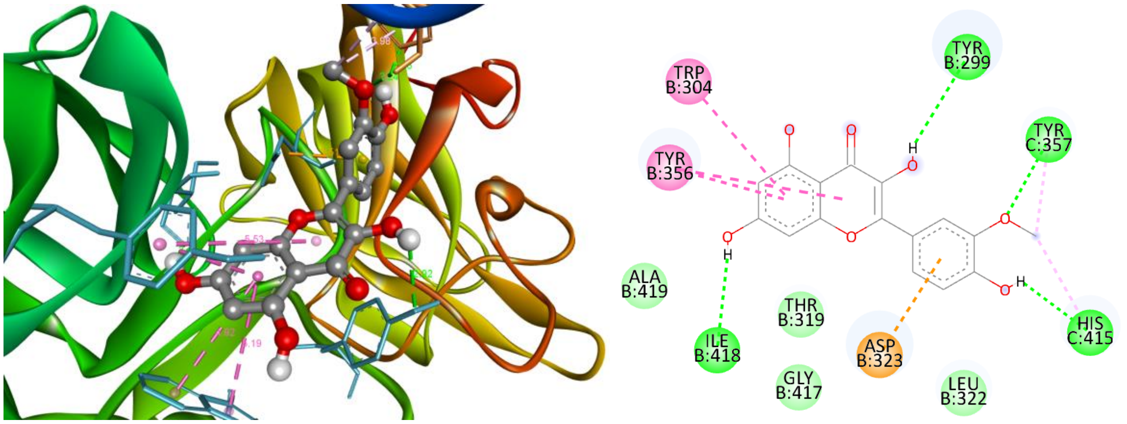

5DN2_VEGF-A VS 72

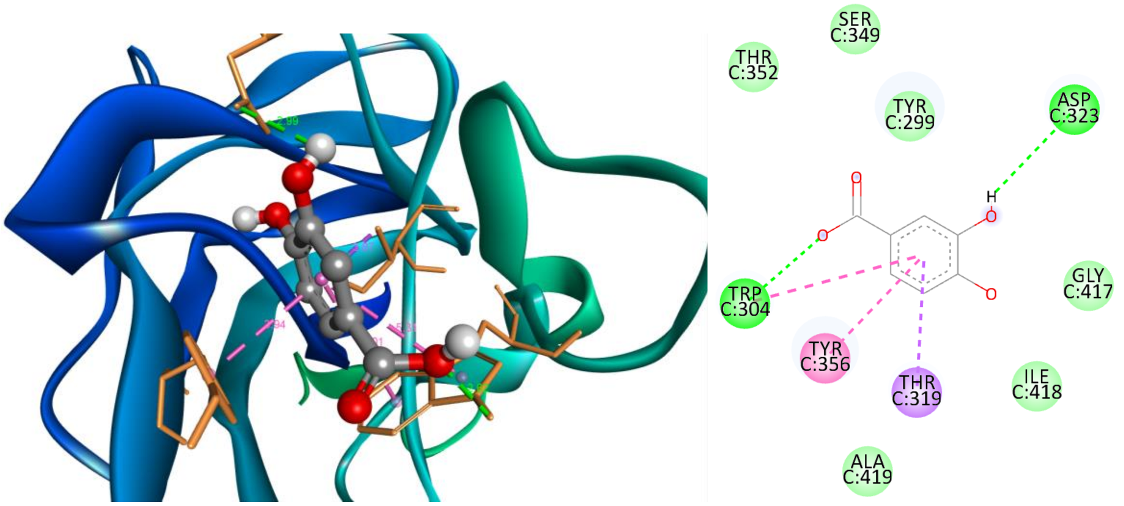

Supplementary Figure 3. Two-Dimensional and three-Dimensional view of hydrogen and hydrophobic bonds interaction of selected top hit hub proteins (based on sub-network) docked with FBR-1741 variety guided by network pharmacology analysis top correlated metabolites (ferulic acid, quercetin, isorhamnetin, protocatechuic acid, and irisflorentin). Note: The legend of the 2D interaction view shows that dark green represents conventional hydrogen bonds, milky green represents hydrophobic interactions, and Pink represents Pi-Alkyl group interacting residues.

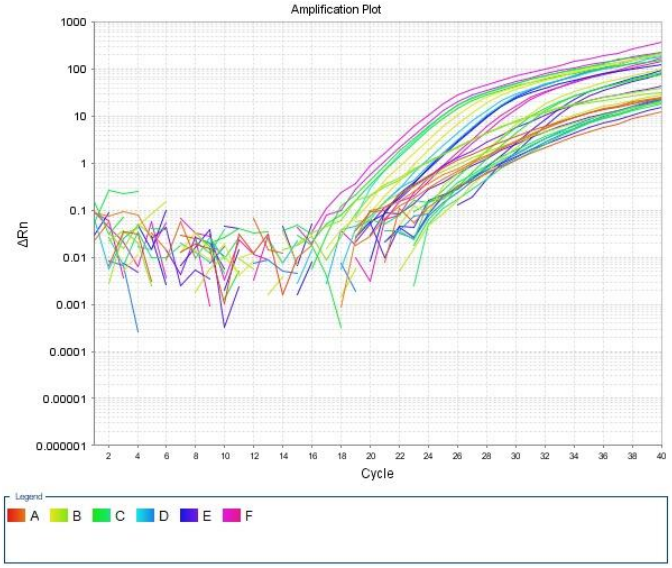

(a)

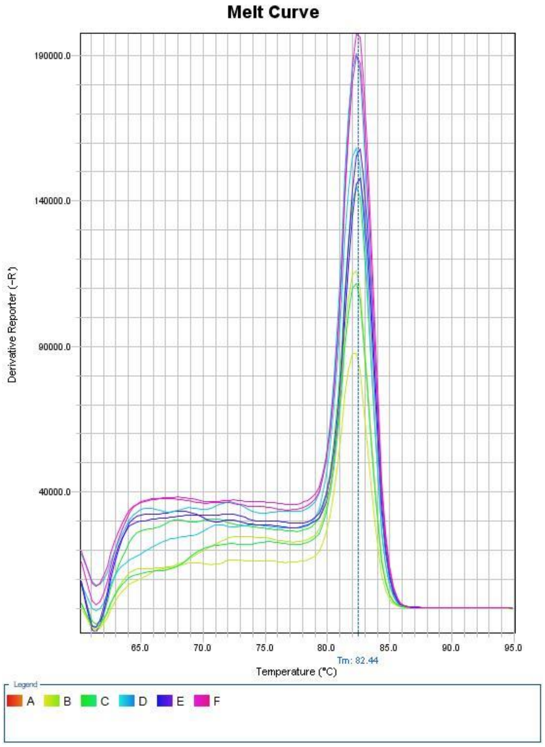

(b)

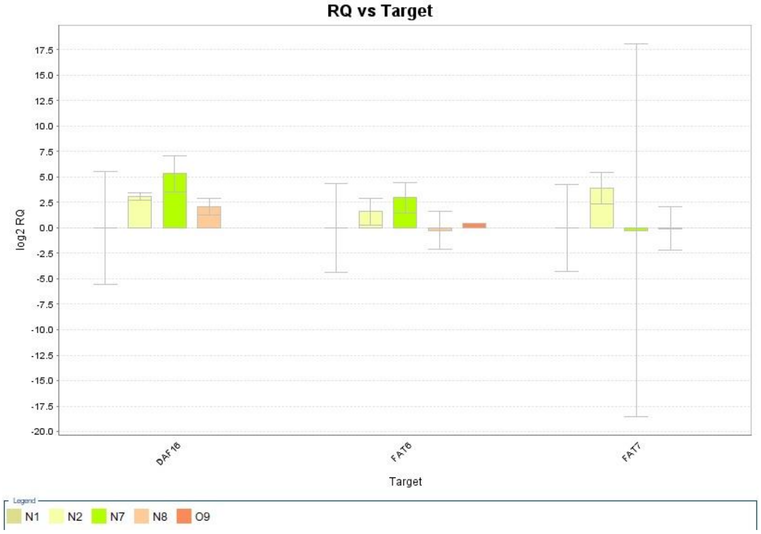

(c)

Supplementary Figure 4. The FBR-1741 and metabolites treatments using qPCR method; (a) Amplification plot; (b) Melt curve peaks for detecting fat genes in *C. elegans* models; (c) Plots of logarithmic RQ values showing the time-course of the expression of fat genes for FBR and metabolite treatments.

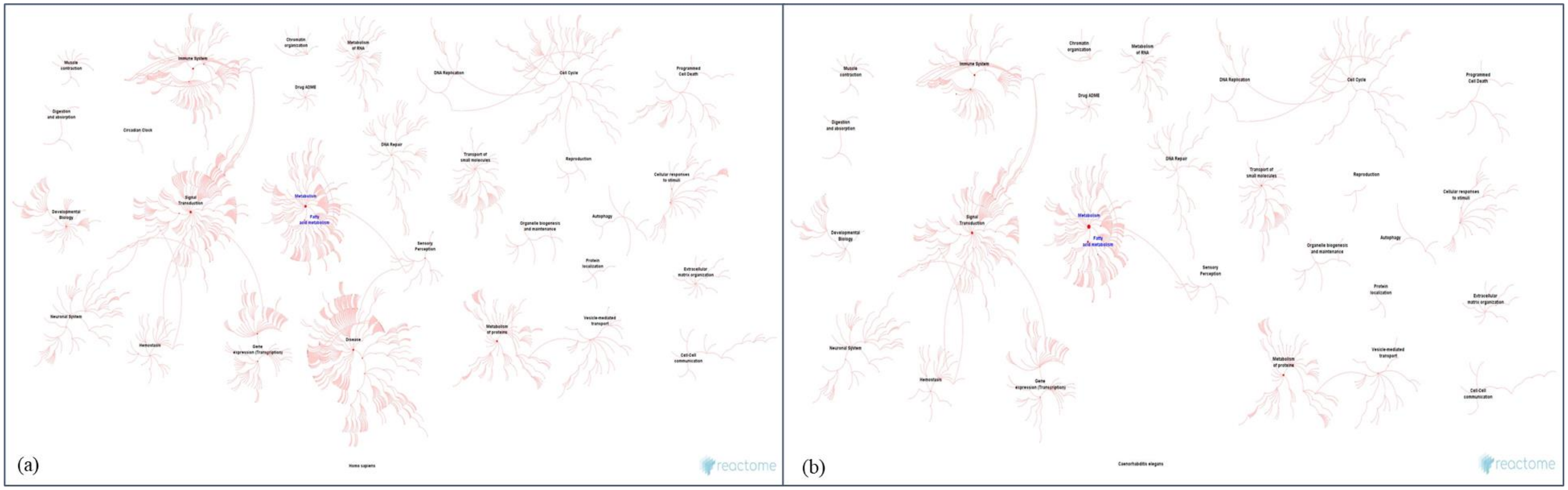

Supplementary Figure 5. Fat synthesis and breakdown processes are controlled by Dietary polyphenols; such can be recognized in fermented brown rice. The pathways show similarity to the animal model used in our study. a), Human metabolic pathway; b) *C. elegans* metabolic Pathways; Processes annotated in this module include the synthesis of fatty acids from acetyl-CoA, mitochondrial and peroxisomal breakdown of fatty acids, and the metabolism of eicosanoids and related molecules.

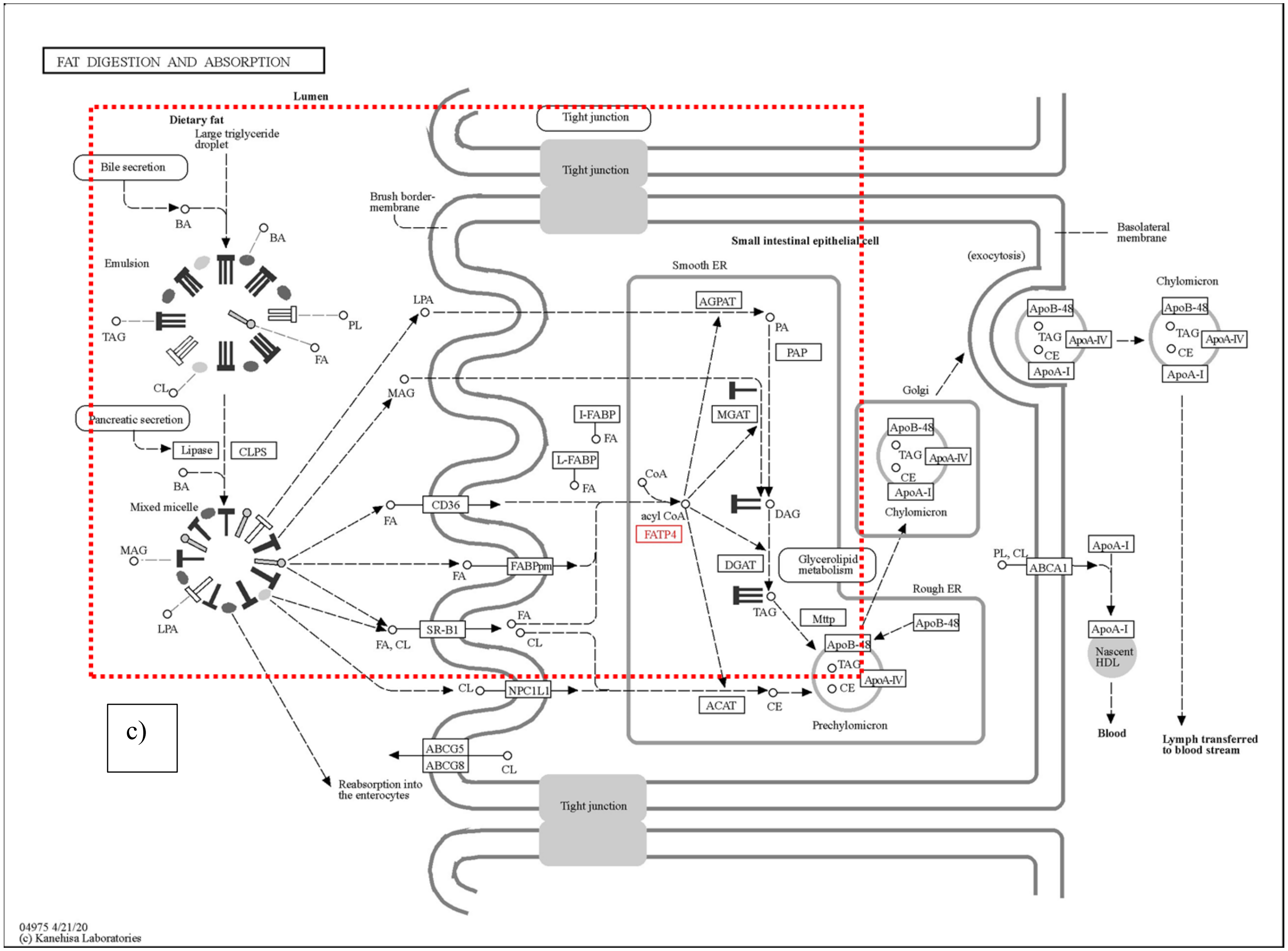

Supplementary Figure 5c. Fat is an important energy source from food. More than 95% of dietary fat is long chain triacylglycerols (TAG), the remaining being phospholipids (4.5%) and sterols. In the small intestine lumen, dietary TAG is hydrolyzed to fatty acids (FA) and monoacylglycerols (MAG) by pancreatic lipase.

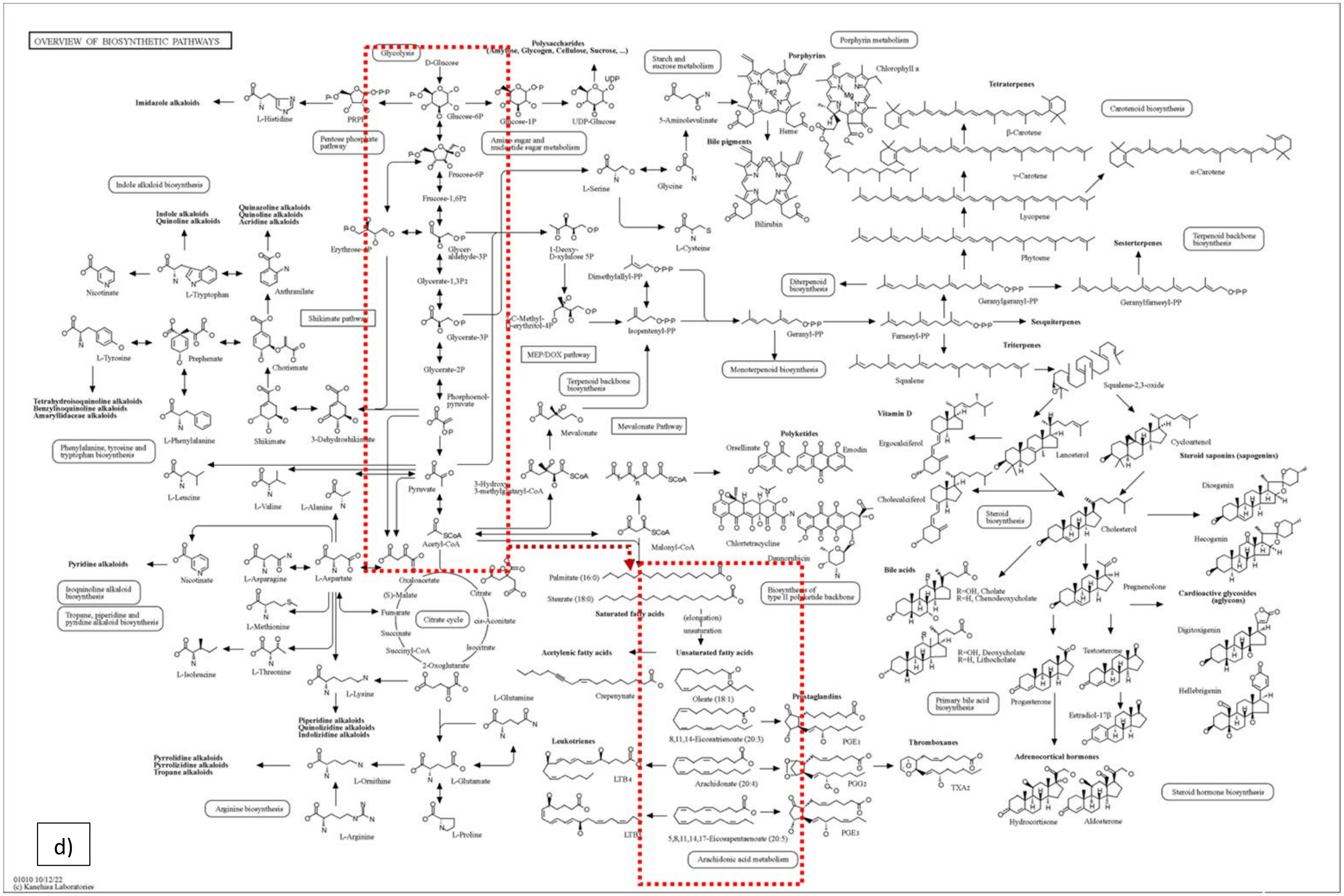

Supplementary Figure 5d. Overview of Biosynthesis Pathway. The red color doted box indicates the formation and degradation of PUFA.

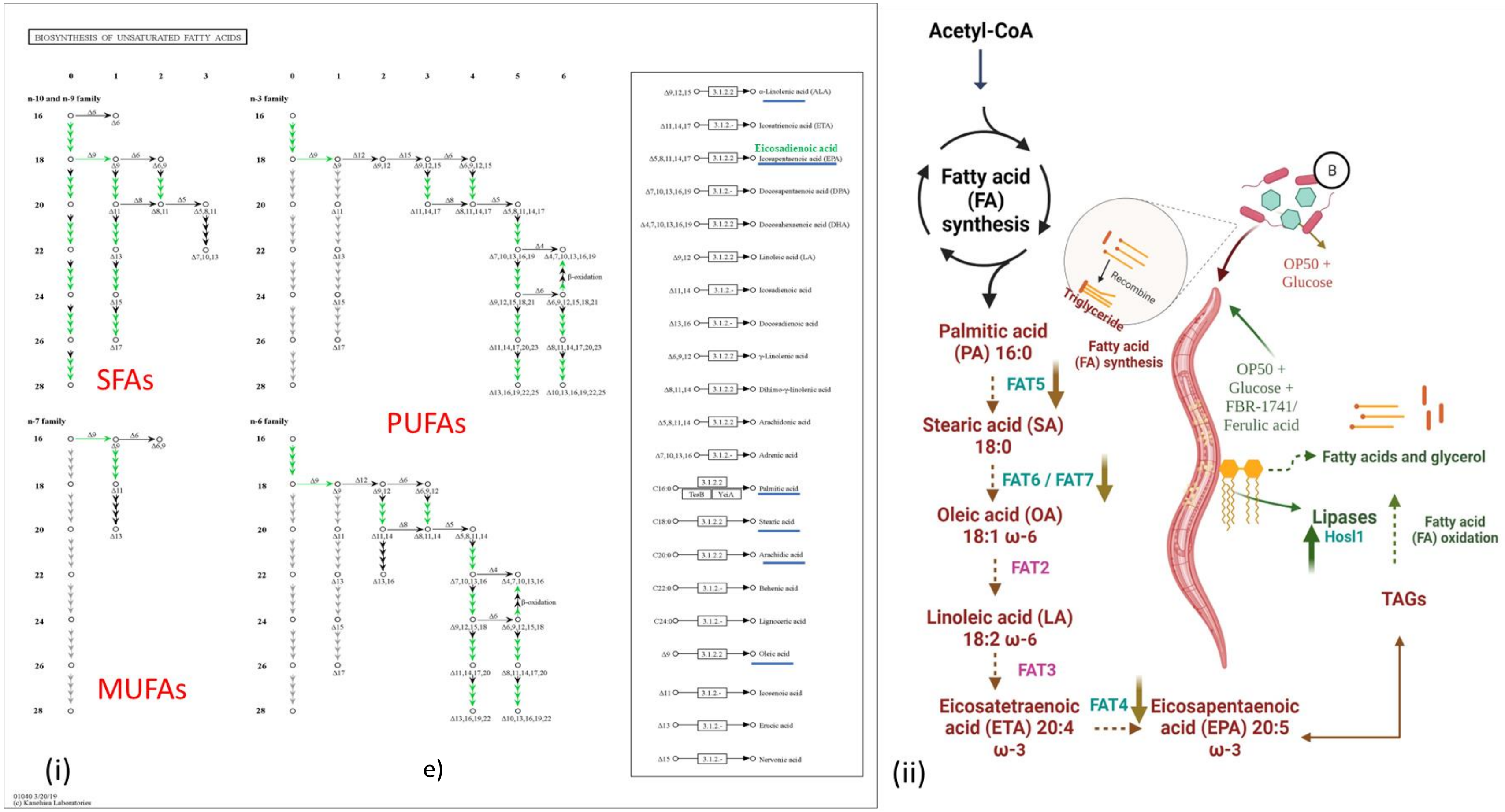

Supplementary Figure 5e. Formation and degradation of PUFA. Starting from the essential Fatty acids oleic acid, linoleic acid, and linolenic acid, the various (n-9)-, (n-6)- and (n-3)-PUFA are formed in sequential steps (at the level of CoA-ester); the Blue color line indicates *C. elegans* metabolomics identified metabolites (ii) Schematic representation highlighting the relationship between fatty acid synthesis and fatty acid oxidation in *C. elegans*, The FBR-1741 and Ferulic acid initiated the enzyme activities of implicated genes are indicated beside brown arrow, in red font to indicate that transcripts encoding that enzyme are downregulated with increasing life span, or in green to show upregulation. Lipids increase in melting temperature with increasing chain length and/or saturation level; thus, shift toward shorter chains with less desaturation, as seen in long-lived *C. elegans*.

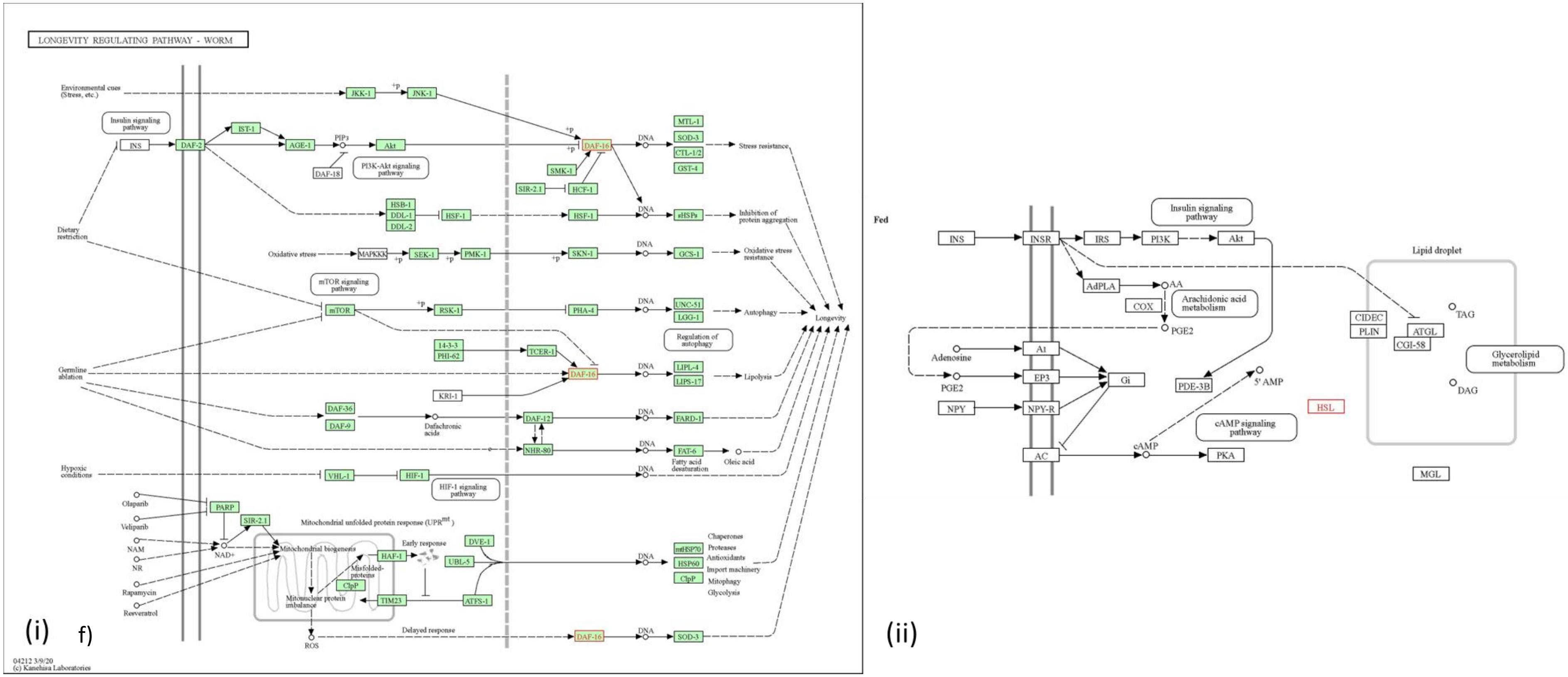

Supplementary Figure 5f. The nematode *Caenorhabditis elegans* has 70 genes that have been found to influence lifespan in this worm. Lifespan extension via Dietary restriction (FBR-1741), germline and other conditions depends on at least four signaling mechanisms: reduced TOR signaling, DAF-16/FOXO regulation, increased steroid signaling via the DAF-36/DAF-9/DAF-12 pathway, and increased NHR-80/HNF-4 signaling; (ii) Lipolysis in adipocytes, the hydrolysis of triacylglycerol (TAG) to release fatty acids (FAs) and glycerol for use by other organs as energy substrates, is a unique function of white adipose tissue. Lipolysis is under tight hormonal control. PKA phosphorylates target proteins such as hormone-sensitive lipase (HSL) and perilipin 1 (PLIN). PLIN phosphorylation is a key event in the sequential activation of TAG hydrolysis involving adipose triglyceride lipase (ATGL), HSL, and monoglyceride lipase (MGL). During the FBR-1741 and Ferulic acid-fed state, insulin, through activation of phosphodiesterase-3B (PDE-3B), inhibits catecholamine-induced lipolysis via the degradation of cAMP.
